# The Poisson CUSUM Chart for Monitoring Small Counts: Addressing the Estimation Uncertainty

**DOI:** 10.1002/bimj.70127

**Published:** 2026-04-05

**Authors:** Stan Heidema, Ivo V. Stoepker, Ralph Huits, Edwin R. van den Heuvel

**Affiliations:** ^1^ Department of Mathematics and Computer Science Technische Universiteit Eindhoven Eindhoven The Netherlands; ^2^ Department of Infectious Tropical Diseases and Microbiology IRCCS Sacro Cuore Don Calabria Hospital Negrar di Valpolicella, (VR) Italy

**Keywords:** conditional performance, control charts, count data, disease surveillance, parameter estimation

## Abstract

This study addresses the impact of estimating in‐control parameters on the performance of the Poisson cumulative sum (CUSUM) chart in detecting an increase in the mean when the counts are small. To reduce false signals induced by estimation uncertainty, recommendations prescribe impractically large sample sizes or propose methods that widen control limits. However, these methods are ineffective for small Poisson counts. We describe three approximate methods from the literature and introduce a novel exact approach that requires no heuristics or approximations, ensuring guaranteed in‐control performance. The theoretical foundation behind this approach is generalizable to other distributions, such as other one‐parameter distributions of the exponential family. Simulations confirm that existing methods fail to achieve the desired in‐control performance, unlike the exact approach. We offer recommendations on how domain knowledge can often be incorporated into the exact approach to improve out‐of‐control performance. Finally, we validate the practical effectiveness of our proposed method through retrospective monitoring of chikungunya infections among returning international travelers reported to the GeoSentinel network. In our case study, the knowledge‐based exact method successfully detects nine major outbreaks at the cost of only one false alarm. Existing methods show a lower positive predictive value for predicting major outbreaks.

## 1 Introduction

Parametric control charts, such as the cumulative sum (CUSUM) chart (Page 1961), are powerful tools for detecting small shifts in processes within diverse domains, including industry (Woodall and Montgomery 2014) and public health (Hutwagner et al. 2003). In settings where in‐control parameters are unknown, a two‐phase procedure involving Phase I and Phase II is often followed. In Phase I, the goal is to precisely characterize the in‐control process by estimating its parameters. The process is assumed to be in‐control throughout the entire duration of Phase I, or at least contains enough information to determine an in‐control setting. Subsequently, Phase II involves monitoring observations to detect changes in the process from the in‐control model.

Finite Phase I sample sizes introduce uncertainty in parameter estimation, which, if unaccounted for, can lead to excessive false alarms due to inaccurate control limits (Jensen et al. 2006; Psarakis et al. 2014). Specifically, for the Poisson CUSUM chart (Lucas 1985) with plugged‐in parameter estimates, a recommendation of Phase I sample sizes greater than or equal to 200 is made for satisfactory in‐ and out‐of‐control performance (Testik 2007). Given the impracticality of such large sample sizes, bootstrap‐based methods have been proposed that aim to guarantee a guaranteed in‐control performance (GICP) condition for smaller sample sizes (Jones and Steiner 2012; Gandy and Kvaløy 2013). In essence, the GICP condition ensures that in‐control performance measures are attained with high probability.

However, existing methods are based on approximations such that the GICP condition is only approximately attained. These approximations are particularly crude if the in‐control mean count is small or the Phase I sample size is small. We are particularly interested in the context of disease surveillance, wherein both of these complicating circumstances may be present (see, e.g., our case study in Section 6). Therefore, using the existing methodology in such contexts may be problematic as these can lead to an inflation of false alarms. This, in turn, may cause substantial long‐term negative consequences, such as economic impacts following travel restrictions, or public mistrust in the validity of the alarms (Nicola et al. 2020). Motivated by these challenges, we aim to adjust the control limits of the Poisson CUSUM chart to exactly satisfy the GICP condition.

Jones and Steiner (2012) introduced a bootstrap method, now the de facto standard (Capizzi and Masarotto 2020), for approximately addressing estimation uncertainty in CUSUM charts, to manage estimation errors within risk‐adjusted binary CUSUM charts. Gandy and Kvaløy (2013) elaborated on this method, providing an asymptotic guarantee regarding the GICP condition. Saleh et al. (2016) illustrated the effect of controlling in‐control conditional performance on out‐of‐control performance in CUSUM charts through this bootstrap method. Furthermore, the robustness of this approach in the presence of autocorrelation has been explored by Weiß et al. (2018). The method is also recommended in Shewhart‐like charts such as $S^{2}$‐charts (Faraz et al. 2015) and np‐charts (Faraz et al. 2017). Additionally, Zhao and Driscoll (2016) proposed an alternative approximate approach based on quantiles of the bootstrap distribution for monitoring Poisson count data using a c‐chart, a Shewhart‐like chart for count data, which can be extended to the Poisson CUSUM chart.

To our knowledge, there are no methods that attain the GICP condition exactly for CUSUM charts in this context. Apart from the approximate nature of existing methodologies, these methods are especially inadequate in scenarios where all Phase I observations are zero due to the degeneracy of the bootstrap distribution on which they are based. This gap motivates our work, which focuses on developing a methodology to attain exact compliance with the GICP condition while effectively addressing the scenario where all Phase I observations are zero.

*Contributions:* Our main contribution is the proposal of a novel exact approach that guarantees the GICP condition for all possible parameter settings without requiring heuristic adjustments. This approach is applicable not only to Poisson CUSUM charts but also to any distribution where an order on their parameters implies stochastic dominance, such as one‐parameter distributions of the exponential family. We also provide recommendations for tuning the exact approach by incorporating domain knowledge, which can improve out‐of‐control performance while maintaining exact GICP adherence.

We describe how to apply the two main methods used in the literature—bootstrap (Jones and Steiner 2012; Gandy and Kvaløy 2013) and approximate bootstrap (Weiß et al. 2018)—to adjust control limits in the context of Poisson CUSUM charts. Additionally, we demonstrate how to extend the quantile method of Zhao and Driscoll (2016) to Poisson CUSUM charts. To enable comparisons in scenarios that may result in all Phase I observations being zero, we provide a heuristic, as these methods otherwise fail to adjust for estimation uncertainty completely.

We compare all approaches through simulation studies, highlighting their differences in in‐control and out‐of‐control performance. To support practitioners, we supply R code for all described methods.

Finally, we highlight the practical relevance of the knowledge‐based exact method through a case study on chikungunya outbreaks among international travelers from 2017 to 2023, using data from the GeoSentinel network. The method successfully identifies nine major outbreaks with only one false alarm. The other methods, however, exhibited higher false alarm rates, consistent with our simulation results.

*Organization:* Section 2 introduces the Poisson CUSUM chart under the assumption of known parameters. In Section 3, we address issues arising from parameter estimation, including scenarios where all Phase I observations are zero (Section 3.1) and violations of in‐control performance guarantees (Section 3.2). Section 3.3 simulates the performance of the Poisson CUSUM chart when the in‐control mean is estimated from a finite Phase I sample, and unadjusted limits are employed. In Section 3.4, we review existing methods to adjust control limits—bootstrap, approximate bootstrap, and quantile method—before introducing our novel exact method in Section 4, along with recommendations for improving out‐of‐control performance by incorporating domain knowledge. Section 5 presents a simulation study comparing the in‐ and out‐of‐control performance of these methods. We apply our methodology to chikungunya disease surveillance in Section 6 and conclude with a discussion in Section 7.

## 2 The Poisson CUSUM Chart With Known Parameters

We start by introducing the Poisson CUSUM chart along with some useful notation. Assume that the (Phase II) observations $Y=\left(Y_{1},Y_{2},\text{…}\right)\overset{\text{i.i.d.}}{∼}P_{\lambda }$ are independent Poisson random variables with parameter λ, respectively. The monitored process is assumed to be in‐control when $\lambda =\lambda_{0}$, for some in‐control rate $\lambda_{0}$. All values are observed at regular time intervals. We focus on a one‐sided chart to detect an increase in the mean (i.e., $\lambda >\lambda_{0}$). The out‐of‐control parameter $\lambda_{1}$ is parameterized as an additive shift of L standard deviations from the in‐control parameter $\lambda_{0}$, a common approach in epidemic shift models (e.g., Hutwagner et al. 2003; Pervaiz et al. 2012). Specifically, we define $\lambda_{1}=\lambda_{0}+L\sqrt{\lambda_{0}}$, where L>0. Although this parameterization is explicitly chosen, other types of out‐of‐control characterizations, such as two‐sided charts or multiplicative shifts (as in Höhle and Paul 2008), can also be incorporated into the methods discussed in this work.

To monitor this increase, the CUSUM statistic is defined recursively as $C_{t}\left(k\right)=\max\left(0,C_{t-1}\left(k\right)+Y_{t}-k\right)$ , where $C_{0}\left(k\right)=0$, and k≥0 is a reference value (Lucas 1985). A standard choice for this reference value is given by

(1) $$\begin{array}{c} k_{\lambda_{0}}=\frac{\lambda_{1}-\lambda_{0}}{\log\left(\lambda_{1}/\lambda_{0}\right)}=\frac{L\sqrt{\lambda_{0}}}{\log\left(1+L\;/\sqrt{\lambda_{0}}\right)}\;, \end{array}$$

which aligns the CUSUM statistic $C_{t}\left(k\right)$ with a scaled version of the Wald sequential probability ratio test statistic (Wald 1945), resulting in optimal power properties (Lucas 1985).

The in‐control hypothesis is rejected whenever the CUSUM chart exceeds a control limit, which we denote by h. Denote the first time at which the chart signals a change (commonly referred to as the run length) by $\tau \left(k,h\right)=\min\left\{t\in N^{+}:C_{t}\left(k\right)>h\right\}$. The average run length (ARL) for a given Phase II distribution P is then defined as the expectation of the run length, that is, $\text{ARL}\left(P,k,h\right)=E_{Y∼P}\left[\tau \left(k,h\right)\right]$. Subsequently, the control limit h is chosen to ensure that the procedure attains a prespecified nominal in‐control average run length of γ. Specifically,

(2) h(P,k)=inf{h≥0:ARL(P,k,h)≥γ}

denotes the smallest control limit guaranteeing an ARL of (at least) γ, with an in‐control Phase II distribution P, and the reference value given by k. In the notation, we suppress the dependence of the control limit on γ. Note that our prescribed γ serves as a lower bound in the above definition due to the discrete nature of the Poisson CUSUM chart, which may make the exact in‐control γ unattainable. Calibrating the control limits h to ensure (2) can, for instance, be done through Monte Carlo simulations. However, in this work, the more efficient Markov chain techniques (Brook and Evans 1972), as implemented in the spc package in R version 4.4.0, are employed (Knoth 2022; R Core Team 2023).

## 3 The Poisson CUSUM Chart With Estimated Parameters

In Section 2, we discussed the Poisson CUSUM chart under the assumption that the in‐control parameter $\lambda_{0}$ is known. This parameter is crucial for two aspects: the computation of the CUSUM statistic (via the reference value $k_{\lambda_{0}}$ in (1)) and the calibration of the in‐control ARL through the control limits in (2).

In the absence of knowledge of $\lambda_{0}$, a natural approach is to use observations from Phase I to compute these quantities. Let the Phase I sample size be $m\in N^{+}$ and assume that the Phase I observations $X=\left(X_{1},\text{…},X_{m}\right)\overset{\text{i.i.d.}}{∼}P_{\lambda_{0}}$ are independent Poisson random variables with parameter $\lambda_{0}$. Based on these data, the reference value and control limit are computed as functions of X, denoted by k(X) and h(X). Specifically, the maximum likelihood estimate (MLE), ${\hat{\lambda }}_{0}^{\text{MLE}}=\frac{1}{m}\sum_{i=1}^{m}X_{i}$, can be “plugged in” both into the reference value and the Phase II distribution, so that

(3) $$k\left(X\right)=k_{{\hat{\lambda }}_{0}^{\text{MLE}}},\;h\left(X\right)=h\left(P_{{\hat{\lambda }}_{0}^{\text{MLE}}},\;k_{{\hat{\lambda }}_{0}^{\text{MLE}}}\right).$$

While this “plugged‐in” approach is intuitive, it leads to two complications: first, it leads to a degenerate chart design when ${\hat{\lambda }}_{0}^{\text{MLE}}=0$ (discussed in Section 3.1), and, more importantly, it introduces variability in the in‐control ARL performance (discussed in Section 3.2).

### 3.1 Heuristic Approach When All Phase I Observations Are Zero

When all observations in the Phase I period are zero (i.e., ${\hat{\lambda }}_{0}^{\text{MLE}}=0$), the CUSUM chart with “plugged‐in” parameters defined in (3) becomes degenerate. Although the optimal reference value can be defined as zero, given that $\lim_{\lambda_{0}↓0}k_{\lambda_{0}}=0$ (cf. Equation 1), simulation from the estimated distribution $P_{0}$ remains necessary for calculating the control limit $h(P_{0},0)$. As $P_{0}$ is degenerate at 0, Phase I run lengths are infinite for all control limits h≥0, so the calibrated control limit is $h(P_{0},0)=0$. During Phase II, if $Y∼P_{\lambda }$, with both the reference value and control limit set to zero, any nonzero observation triggers an alarm, resulting in a degenerate chart with an ARL of $\frac{1}{1-\;e^{-\lambda }}$, the expected time until a nonzero observation occurs.

In this scenario, existing methods that aim to approximately account for estimation uncertainty (defined later in this section) fail to do so and lead to the same degenerate chart as one would obtain with the plugged‐in estimator. In practice, this is not problematic when $\lambda_{0}$ and m are such that $P_{X∼P_{\lambda_{0}}}\left[\sum_{i=1}^{m}X_{i}=0\right]=e^{-m\lambda_{0}}$ is sufficiently small. However, in the scenarios of interest, all‐zero Phase I sets are not uncommon. In these cases, a heuristic approach can be employed by slightly modifying the classical maximum likelihood estimator as

(4) $$\begin{array}{c} {\hat{\lambda }}_{0}=\left\{\begin{array}{cc} \frac{0.5}{m} & \;\text{if}\;\sum_{i=1}^{m}X_{i}=0\\ \frac{1}{m}\sum_{i=1}^{m}X_{i} & \;\text{if}\;\sum_{i=1}^{m}X_{i}>0 \end{array}\right.\;. \end{array}$$

This heuristic introduces a bias in the estimator but allows existing approximate adjustment methods to produce nondegenerate adjusted CUSUM charts in cases of an all‐zero Phase I set, facilitating comparisons across methodologies in regimes where all‐zero Phase I sets may occur. Given this estimator (4), we define the *unadjusted control limit* as $h(P_{{\hat{\lambda }}_{0}},k_{{\hat{\lambda }}_{0}})$.

### 3.2 Guaranteed In‐Control Performance

Estimation errors can induce variability in the in‐control performance guarantees of the chart. Specifically, this variability appears in the distribution of the conditional average run length

(5) $$\begin{array}{c} \text{CARL}\left(k\left(X\right),h\left(X\right),X,\lambda_{0}\right)=E_{Y∼P_{\lambda_{0}}}\left[\tau \left(k(X),h(X)\right)\;|\;X\right], \end{array}$$

which quantifies the expected time a change is signaled when the chart is calibrated using the Phase I data X, while the true Phase II distribution is $P_{\lambda_{0}}$. In particular, using unadjusted control limits leads to significant deviations of $\text{CARL}\left(k_{{\hat{\lambda }}_{0}},h\left(P_{{\hat{\lambda }}_{0}},k_{{\hat{\lambda }}_{0}}\right),X,\lambda_{0}\right)$ from the desired average run length γ, especially when the Phase I sample size m is small (Jensen et al. 2006; Psarakis et al. 2014). To mitigate the effect of this variability, it is crucial to construct control limits h(X) such that the conditional average run length (5) is, with high probability, at least the prescribed value γ. This concept, known as GICP, is defined as follows (Jones and Steiner 2012; Gandy and Kvaløy 2013):

(6) $$\begin{array}{c} \underset{\lambda_{0}>0}{\inf}P_{X∼P_{\lambda_{0}}}\left[\text{CARL}\left(k\left(X\right),h\left(X\right),X,\lambda_{0}\right)\geq \gamma \right]=1-\alpha , \end{array}$$

where α∈(0,1), representing a sort of Type I error due to estimation uncertainty, is small.

### 3.3 Conditional in‐Control Performance of the Poisson CUSUM Chart With Estimated Parameters

We investigate the conditional performance of the Poisson CUSUM chart when its parameter is estimated from a finite Phase I sample of size m, and unadjusted control limits are employed. To this end, we calculate the average (conditional) average run length under unadjusted control limits, which is given by

(7) $$\begin{array}{cc} \text{AARL}(\lambda_{0},\lambda ) & =E_{X∼P_{\lambda_{0}}}\left[\text{CARL}\left(k_{{\hat{\lambda }}_{0}},h\left(P_{{\hat{\lambda }}_{0}},k_{{\hat{\lambda }}_{0}}\right),X,\lambda \right)\right], \end{array}$$

capturing the expected behavior of the conditional average run length (5) under estimation uncertainty in $\lambda_{0}$. We refer to the in‐control and out‐of‐control quantities as ${\text{AARL}}_{0}=\text{AARL}\left(\lambda_{0},\lambda_{0}\right)$ and ${\text{AARL}}_{1}=\text{AARL}\left(\lambda_{0},\lambda_{1}\right)$, respectively, following Saleh et al. (2016) and Zhao and Driscoll (2016). Additionally, since the conditional average run length is a random variable subject to uncertainty, its variance, which quantifies the variability resulting from estimation uncertainty, is relevant and defined as

(8) $$\begin{array}{c} \begin{array}{cc} \text{VARL}(\lambda_{0},\lambda ) & =E_{X∼P_{\lambda_{0}}}\left[\text{CARL}{\left(k_{{\hat{\lambda }}_{0}},h\left(P_{{\hat{\lambda }}_{0}},k_{{\hat{\lambda }}_{0}}\right),X,\lambda \right)}^{2}\right]\\ & \;-\text{AARL}{\left(\lambda_{0},\lambda \right)}^{2}. \end{array} \end{array}$$

Again, we write ${\text{VARL}}_{0}=\text{VARL}\left(\lambda_{0},\lambda_{0}\right)$. Finally, to provide comparisons across different settings, we normalize the variance and report the relative standard deviation of the conditional average run length, ${\text{RSDARL}}_{0}$, defined as

(9) $$\begin{array}{c} {\text{RSDARL}}_{0}=\frac{\sqrt{{\text{VARL}}_{0}}}{{\text{AARL}}_{0}}. \end{array}$$

**FIGURE 1:**
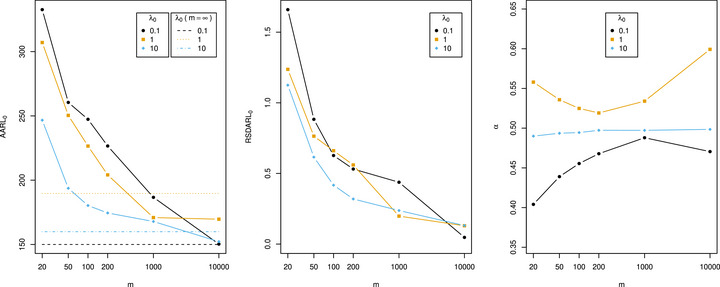
Conditional in‐control performance metrics with respect to the Phase I sample size m under unadjusted limits for various in‐control rates $\lambda_{0}$ are presented. Parameters are selected as $\lambda_{0}\in \left\{0.1,1,10\right\}$, L=2, and $m\in \left\{20,50,10^{2},2\cdot 10^{2},10^{3},10^{4},\infty \right\}$, with m=∞ representing the scenario where the parameter is known. The minimum in‐control average run length is set to γ=150. A rational approximation with three digits of precision after the decimal point is chosen, and $10^{4}$ simulation runs for both Phase I and Phase II are conducted. The left figure shows the in‐control average conditional average run length (${\text{AARL}}_{0}$). Here, dotted lines indicate the achieved $\text{ARL}\left(P_{\lambda_{0}},k_{\lambda_{0}},h\left(P_{\lambda_{0}},k_{\lambda_{0}}\right)\right)$ values for m=∞, and may exceed γ=150 due to the discreteness of the chart. The middle figure illustrates the relative standard deviation of the in‐control conditional average run length (${\text{RSDARL}}_{0}$). The right figure shows the probability α that the conditional average run length is smaller than the nominal average run length γ. The *x*‐axes are on a log scale.

Figure 1 presents the conditional in‐control performance metrics ${\text{AARL}}_{0}$ (Equation 7), ${\text{RSDARL}}_{0}$ (Equation 9), and α (Equation 6), respectively. Testik (2007) recommends to have Phase I sample sizes of at least 200 to achieve in‐control performance close to that of known parameters. However, such recommendations should not be made without considering the in‐control parameter, $\lambda_{0}$. Specifically, lower values of $\lambda_{0}$ lead to higher standard deviations. As highlighted by Figure 1, for m≤200, it is evident that calibrating control limits under the assumption of no estimation uncertainty leads to an inflation of ${\text{AARL}}_{0}$, with relative standard deviations ranging from 0.32 to 1.66. Notably, even for m=1000 and $\lambda_{0}=0.1$, the relative standard deviation (0.44) is still substantial. This shows that increasing the sample size does not effectively address the issue when dealing with a small in‐control rate. Finally, the observed α values, which range from 0.40 to 0.60 across all examined scenarios, suggest that the GICP condition (6) cannot be reliably met for any practical choices of α when using unadjusted limits.

### 3.4 Existing Methods to Adjust Control Limits for the Poisson CUSUM Chart

As shown in the previous section, employing unadjusted limits under estimation uncertainty results in undesirable conditional performance, especially when the Phase I sample size, or the mean count, is small. To satisfy the GICP condition (6), various methods have been developed to calculate more conservative control limits in the presence of estimation uncertainty. In this section, we discuss three methods of this kind: the bootstrap method, the approximate bootstrap method, and the quantile method.

The following *bootstrap method*, based on parametric bootstrapping, is initially described by Jones and Steiner (2012) and Gandy and Kvaløy (2013), and used in various contexts (e.g., Saleh et al. 2016). The procedure is currently considered the recommended method of dealing with estimation uncertainty within control charts (Capizzi and Masarotto 2020):

1. Given m Phase I observations $X_{1},\text{…},X_{m}$ from the assumed true process distribution $P_{\lambda_{0}}$, estimate the in‐control process parameter ${\hat{\lambda }}_{0}$.
2. Generate m bootstrap samples of Phase I data from the bootstrap distribution $P_{{\hat{\lambda }}_{0}}$. Compute the bootstrap estimate ${\hat{\lambda }}^{*}$ using Equation (4). Repeat B times to get ${\hat{\lambda }}_{1}^{*},\text{…},{\hat{\lambda }}_{B}^{*}$.
3. Let $q_{\alpha }^{*}$ be the α empirical lower quantile of $\left\{h\left(P_{{\hat{\lambda }}_{b}^{*}},k_{{\hat{\lambda }}_{b}^{*}}\right)-h\left(P_{{\hat{\lambda }}_{0}},k_{{\hat{\lambda }}_{b}^{*}}\right):b=1,\text{…},B\right\}$.
4. The adjusted control limit is set as $h^{*}=h\left(P_{{\hat{\lambda }}_{0}},k_{{\hat{\lambda }}_{0}}\right)-q_{\alpha }^{*}$.

This procedure is popular because of the following asymptotic optimality property, as proved by Gandy and Kvaløy (2013):

(10) $$\begin{array}{c} \lim_{m\to \infty }P_{X∼P_{\lambda_{0}}}\left[h\left(P_{\lambda_{0}},k_{{\hat{\lambda }}_{0}}\right)\leq h^{*}\right]=1-\alpha \;. \end{array}$$

This property holds under suitable regularity conditions, which are proven to be satisfied by the CUSUM chart with normal observations, as well as by a nonparametric setup for CUSUM charts (Gandy and Kvaløy 2013).

In the Shewhart individuals control chart, under i.i.d. normal data, the control limit under estimation equals the control limit under the bootstrap distribution (Weiß et al. 2018) (which corresponds in our notation to the equivalence: $h\left(P_{{\hat{\lambda }}_{0}},k_{{\hat{\lambda }}_{0}}\right)=h\left(P_{{\hat{\lambda }}_{b}^{*}},k_{{\hat{\lambda }}_{b}^{*}}\right)$ for all b=1,…,B). In other words, the control limits are invariant to the in‐control parameters in this chart. Therefore, an adjusted control limit can also be obtained by estimation of the (1−α)‐quantile of $\left\{h(P_{{\hat{\lambda }}_{0}},k_{{\hat{\lambda }}_{b}^{*}}):b=1,\text{…},B\right\}$, which we denote as ${\tilde{q}}_{1-\alpha }^{*}$, that is, $h_{A}^{*}={\tilde{q}}_{1-\alpha }^{*}$. However, this invariance property is not satisfied in the Poisson CUSUM chart, as $h(P_{{\hat{\lambda }}_{0}},k_{{\hat{\lambda }}_{0}})$ and $h(P_{{\hat{\lambda }}_{b}^{*}},k_{{\hat{\lambda }}_{b}^{*}})$ are not equal for all b=1,…,B. So, this method is not equivalent to the bootstrap procedure and also does not have the same asymptotic guarantee. Still, this method is computationally advantageous as it requires only a single simulation step to calculate $h_{A}^{*}$, compared to the necessary two steps (steps 2 and 3) in the bootstrap procedure. As this approach serves as an approximation to the bootstrap method (Weiß et al. 2018), we refer to this method as the *approximate bootstrap method*.

For the Poisson c‐chart, a Shewhart‐like chart for count data, the control limits are solely based on the desired false alarm rate and the estimated or known mean (Zhao and Driscoll 2016). Therefore, instead of using the bootstrapped sample means to obtain a distribution of the standardized distances, as done for $h^{*}$ and $h_{A}^{*}$, we can use the bootstrap method to obtain a sampling distribution of the estimated mean and then use quantiles from this distribution to adjust the control limits. Similar arguments hold in the Poisson CUSUM chart, where the control limit $h(P_{\lambda_{0}},k_{{\hat{\lambda }}_{0}})$ depends on the nominal average run length γ, the estimated mean ${\hat{\lambda }}_{0}$, and the true mean $\lambda_{0}$. Since we consider upper quantiles of the bootstrapped distribution of the estimated mean, we refer to this method as the *quantile method*:

1. Given m Phase I observations $X_{1},\text{…},X_{m}$ from the assumed true process distribution $P=P_{\lambda_{0}}$, estimate the in‐control process parameter ${\hat{\lambda }}_{0}$.
2. Approximate the distribution of the in‐control parameter estimator ${\hat{\lambda }}_{0}|\lambda_{0}∼\frac{1}{m}P_{m\lambda_{0}}$ by $\frac{1}{m}P_{m{\hat{\lambda }}_{0}}$. Generate B bootstrapped means ${\hat{\lambda }}_{1}^{*},\text{…},{\hat{\lambda }}_{B}^{*}∼\frac{1}{m}P_{m{\hat{\lambda }}_{0}}$ and denote with ${\tilde{q}}_{1-\alpha }$ the (1−α)th quantile of ${\hat{\lambda }}_{1}^{*},\text{…},{\hat{\lambda }}_{B}^{*}$.
3. Estimate the adjusted control limit as $h_{Q}^{*}=h\left(P_{{\tilde{q}}_{1-\alpha }},k_{{\hat{\lambda }}_{0}}\right)$.

Here, the procedure of Zhao and Driscoll (2016) can be simplified computationally by sampling directly from the bootstrapped sampling distribution of ${\hat{\lambda }}_{0}$, although the resulting control limits are equivalent. This method implicitly uses the fact that the control limits are monotone in the bootstrapped means, that is, $h\left(P_{{\hat{\lambda }}_{\left(1\right)}^{*}},k_{{\hat{\lambda }}_{0}}\right)\leq \cdots \leq h\left(P_{{\hat{\lambda }}_{\left(B\right)}^{*}},k_{{\hat{\lambda }}_{0}}\right)$. We state this monotone property more generally as the following lemma:

Lemma 3.1 Let $Y_{1},Y_{2},\text{…}∼F$ and $Y_{1}^{\prime },Y_{2}^{\prime },\text{…}∼F^{\prime }$ be independent univariate random variables such that for all t∈N, $Y_{t}$ is stochastically dominated by $Y_{t}^{\prime }$. Then $h\left(F,k\right)\leq h\left(F^{\prime },k\right)$ for all k≥0.

The proof is available in Supporting Information Appendix B. In the specific context of two Poisson distributions, $P_{\lambda }$ and $P_{\lambda^{\prime }}$, stochastic domination holds if and only if the rate parameters satisfy $\lambda \leq \lambda^{\prime }$. Because of the monotone relationship, and the fact that $P_{X∼P_{\lambda_{0}}}\left[\lambda_{0}\leq {\tilde{q}}_{1-\alpha }\right]\approx 1-\alpha$, the control limit $h_{Q}^{*}$ ensures with probability approximately (1−α) that the GICP condition is met.

## 4 Exact Approaches

A drawback of the existing methods described in Section 3.4 is that approximations are made such that the GICP condition holds only approximately, leading to potentially large deviations from the desired level of α, especially when m or $\lambda_{0}$ is small. To ensure accurate control limits, we propose an approach based on exact confidence intervals, which uniformly guarantees the GICP condition. In other words, we construct a control limit h(X) such that

(11) $$\begin{array}{c} \underset{\lambda_{0}>0}{\inf}P_{X∼P_{\lambda_{0}}}\left[\text{CARL}\left(k\left(X\right),h\left(X\right),X,\lambda_{0}\right)\geq \gamma \right]\geq 1-\alpha , \end{array}$$

The following theorem provides a sufficient condition to uniformly guarantee the GICP condition at level α:

Theorem 4.1 Let $U_{\alpha }$ be an (1−α)·100% upper limit such that

(12) $$\underset{\lambda_{0}\geq 0}{\inf}P\left[\lambda_{0}\leq U_{\alpha }\right]=1-\alpha .$$

Then, for any Phase I data‐dependent reference value k(X)≥0, the CUSUM chart with control limit $h(P_{U_{\alpha }},k\left(X\right))$ uniformly guarantees the GICP condition (11) at level α.

Assume that (12) holds, and let k≥0 be fixed. Then, by Lemma 3.1 we have with at least 1−α probability that

(13) $$h\left(P_{\lambda_{0}},k\right)\leq h\left(P_{U_{\alpha }},k\right)\;\text{for all}\;\lambda_{0}\geq 0.$$

Moreover, under a sequence of Phase II observations distributed according to $P_{\lambda_{0}}$, if control limits satisfy $h\leq h^{\prime }$, then the corresponding run lengths are ordered almost surely, that is, $P_{Y∼P_{\lambda_{0}}}\left[\tau \left(k,h\right)\leq \tau \left(k,h^{\prime }\right)\right]=1$. This implies that whenever $h\leq h^{\prime }$:

(14) $$E_{Y∼P_{\lambda_{0}}}\left[\tau \left(k,h\right)\right]\leq E_{Y∼P_{\lambda_{0}}}\left[\tau \left(k,h^{\prime }\right)\right].$$

Combining (13) and (14), it follows that, with at least probability 1−α, for all $\lambda_{0}\geq 0$:

(15) $$\begin{array}{c} \begin{array}{cc} \gamma & \leq E_{Y∼P_{\lambda_{0}}}\left[\tau \left(k\left(X\right),h\left(P_{\lambda_{0}},k\left(X\right)\right)\right)\;|\;X\right]\\ & \leq E_{Y∼P_{\lambda_{0}}}\left[\tau \left(k\left(X\right),h\left(P_{U_{\alpha }},k\left(X\right)\right)\right)\;|\;X\right]\\ & =\text{CARL}\left(k\left(X\right),h\left(P_{U_{\alpha }},k\left(X\right)\right),X,\lambda_{0}\right). \end{array} \end{array}$$

□

For clarity and concreteness, we present this result specifically for Poisson‐distributed data. However, similarly to Lemma 3.1, Theorem 4.1 can be extended to any distribution where an ordering of parameters implies stochastic dominance. It can be shown that any family of densities $p_{\theta }\left(x\right)$ defined on the real line with a monotone likelihood ratio in x, satisfies this condition (Lehmann and Romano 2005). An important example of such families is the one‐parameter exponential family.

Limits satisfying the condition of Theorem 4.1 are called exact methods, because the infimum over $\lambda_{0}$ of the coverage probability is exactly equal to 1−α (Swift 2009). Exact limits for the Poisson distribution may be conservative for particular choices of $\lambda_{0}$ due to the discrete nature of the distribution. The most well‐known exact upper limit is proposed by Garwood (1936); $U_{\alpha }\left(X\right)=\frac{1}{2m}\chi_{1-\alpha ,2+2\sum_{i=1}^{m}X_{i}}^{-2}$, where $\chi_{1-\alpha ,2+2\sum_{i=1}^{m}X_{i}}^{-2}$ denotes the 1−α quantile of a $\chi^{2}$ distribution with $2+2\sum_{i=1}^{m}X_{i}$ degrees of freedom. We often write $U_{\alpha }=U_{\alpha }\left(X\right)$ for convenience. Garwood upper limits are strictly nested; that is, higher values of α lead to lower upper limits. Although Garwood upper limits are among the most conservative exact upper limits, they are optimal among choices of upper limits preserving the strict nested property (Thulin and Zwanzig 2017).

To construct control limits that satisfy Theorem 4.1, one may employ the Garwood limits and use the maximum likelihood estimate of $\lambda_{0}$ to compute the reference value $k\left(X\right)=k_{{\hat{\lambda }}_{0}^{\text{MLE}}}$, which induces the control limits $h(P_{U_{\alpha }},k_{{\hat{\lambda }}_{0}^{\text{MLE}}})$. We refer to this procedure as the *exact method*.

Note that this procedure does not suffer from calibration issues whenever all Phase I observations are zero, as $U_{\alpha }\left(0\right)>0$. Nonetheless, in this scenario, the CUSUM chart induced by the exact method becomes nondecreasing because the reference value is set to $k_{{\hat{\lambda }}_{0}^{\text{MLE}}}=0$, which necessitates conservative calibration and results in high control limits. Moreover, the design of the chart does not incorporate knowledge of L. Since Theorem 4.1 holds for any Phase I data‐dependent reference value k(X), it is worth investigating whether choices for k(X) exist which may be closer to the optimal (but unknown) value $k_{\lambda_{0}}$ than the plugged‐in value $k_{{\hat{\lambda }}_{0}^{\text{MLE}}}$. In particular, the heuristic estimator defined in (4) may be a suitable alternative. To provide a recommendation for practice, we compare the absolute estimation errors of the two choices when only zeroes are observed and recommend in that case estimating the reference value by $k_{{\hat{\lambda }}_{0}}$ instead of $k_{{\hat{\lambda }}_{0}^{\text{MLE}}}$ whenever its absolute estimator error is smaller, that is,

(16) $$\begin{array}{cc} |k_{\lambda_{0}}-k_{{\hat{\lambda }}_{0}}\left|\leq \right|k_{\lambda_{0}}-k_{{\hat{\lambda }}_{0}^{\text{MLE}}}| & \Leftrightarrow \frac{\sqrt{\lambda_{0}}}{\log\left(1+\frac{L}{\sqrt{\lambda_{0}}}\right)}\\ & \geq \frac{1}{2\sqrt{2m}\log\left(1+L\sqrt{2m}\right)}. \end{array}$$

Solving (16) leads to a minimum value of $\lambda_{0}$ (in terms of L and m) for which, if only zeroes are observed during Phase I, the absolute estimation error of $k_{\lambda_{0}}$ based on the heuristic ${\hat{\lambda }}_{0}$ is smaller than the error based on ${{\hat{\lambda }}_{0}}^{\text{MLE}}$. For a selection of combinations of L and m, these minimum values of the in‐control rate $\lambda_{0}$ are provided in Table 1. For many values of $\lambda_{0}$ of practical interest, using the heuristic estimator ${\hat{\lambda }}_{0}$ reduces the absolute estimation error in $k_{\lambda_{0}}$. The control limit can then be computed as $h(P_{U_{\alpha }},k_{{\hat{\lambda }}_{0}})$. We refer to this approach as the *knowledge‐based exact method*.

**TABLE 1:** Thresholds for which condition (16) holds for different values of L and m. For any $\lambda_{0}$ greater than the threshold, the knowledge‐based exact method is recommended over the exact method.

L∖m	20	50	100	200	1000	10,000
1	$9.31\times 10^{-3}$	$3.59\times 10^{-3}$	$1.75\times 10^{-3}$	$8.55\times 10^{-4}$	$1.64\times 10^{-4}$	$1.56\times 10^{-5}$
2	$8.82\times 10^{-3}$	$3.42\times 10^{-3}$	$1.68\times 10^{-3}$	$8.22\times 10^{-4}$	$1.59\times 10^{-4}$	$1.53\times 10^{-5}$
3	$8.58\times 10^{-3}$	$3.34\times 10^{-3}$	$1.64\times 10^{-3}$	$8.07\times 10^{-4}$	$1.56\times 10^{-4}$	$1.51\times 10^{-5}$

## 5 Simulated Performance of the Poisson CUSUM Chart With Adjusted Control Limits

**FIGURE 2:**
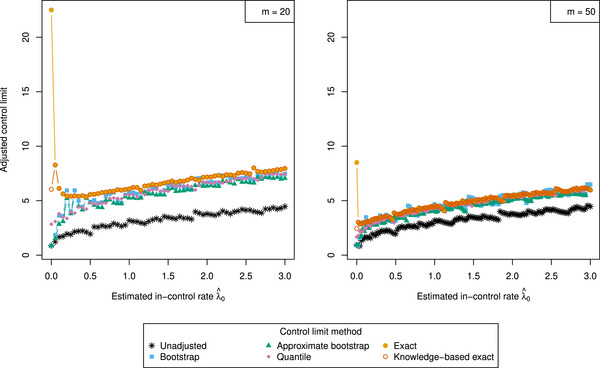
Various control limits with respect to estimated in‐control rates ${\hat{\lambda }}_{0}$ as produced by the unadjusted, bootstrap, approximate bootstrap, quantile, exact, and knowledge‐based exact procedures. Parameter settings are chosen as α=0.05,L=2,γ=150, for m=20 (left) and m=50 (right). The number of bootstrap samples is set to $B=10^{4}$.

In this section, we compare the conditional in and out‐of‐control performance of methodologies to adjust control limits. Figure 2 highlights that the control limits calculated by the different procedures truly deviate from each other, particularly when the estimated in‐control rate is small. To assess in‐control performance, we compare how the methods perform in adhering to the GICP condition (6). Out‐of‐control performance is assessed through the out‐of‐control average conditional average run length (${\text{AARL}}_{1}$, defined in Equation 7). We apply the following simulation procedure (Saleh et al. 2016).

1. Generate m Phase I samples from the assumed process distribution $P_{\lambda_{0}}$. Estimate $\lambda_{0}$ using ${\hat{\lambda }}_{0}$ or ${\hat{\lambda }}_{0}^{\text{MLE}}$, depending on the procedure.
2. Based on the estimate and the corresponding procedure (bootstrap, approximate bootstrap, quantile, exact, or knowledge‐based exact), calculate the adjusted control limit.
3. Set up the CUSUM chart with the initial Phase I estimate and the adjusted control limit. Compute the ARL for the CUSUM chart set with Phase II data from the assumed process distribution $P_{\lambda_{0}}$ for the in‐control ARL and $P_{\lambda_{1}}$ for the out‐of‐control ARL.

To illustrate that the exact method extends to one‐parameter distributions in the exponential family, we also conducted simulations under the binomial distribution with known population size and unknown proportion, as detailed in Supporting Information Appendix C.

### 5.1 In‐Control Performance

**FIGURE 3:**
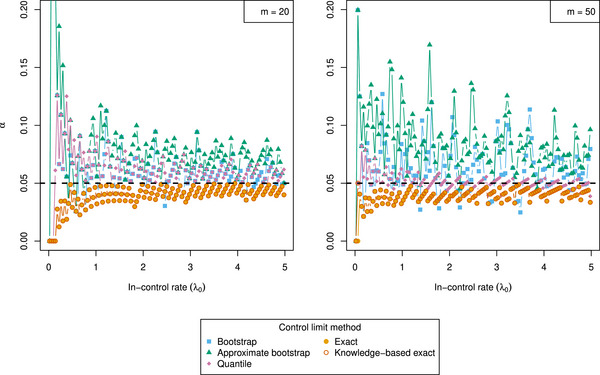
Guaranteed in‐control performance when adjusting control limits according to the bootstrap, approximate bootstrap, quantile, exact, and knowledge‐based exact procedures, with nominal value α=0.05. Remaining parameters are L=2,γ=150, for m=20 (left) and m=50 (right). Steps 1–3 of the simulation procedure are repeated for $N=10^{6}$ times, and the number of bootstrap samples is set to $B=10^{4}$. A rational approximation with three‐digit precision after the decimal point was selected.

We can observe from Figure 3 that attaining GICP adherence is especially difficult for small $\lambda_{0}$. This is particularly visible in the performance of the bootstrap and the approximate bootstrap methods, which do not sufficiently adjust for estimation uncertainty. The effect is also present for the quantile method, although to a lesser degree. This simulation confirms that both exact methods uniformly control the GICP condition, showing nearly identical performance.

### 5.2 Out‐of‐Control Performance

**FIGURE 4:**
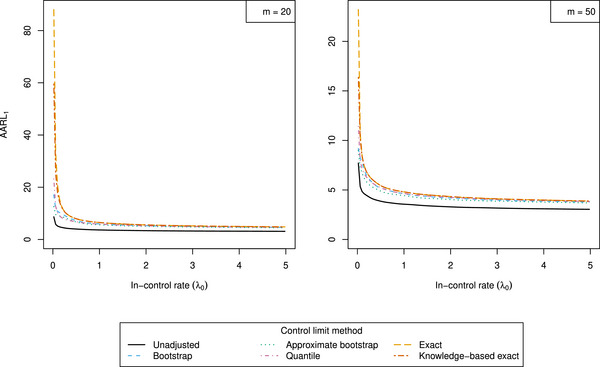
Out‐of‐control average conditional average run length (${\text{AARL}}_{1}$) with respect to true in‐control rate $\lambda_{0}$ when adjusting control limits according to the unadjusted, bootstrap, approximate bootstrap, quantile, exact, and knowledge‐based exact procedures. Parameter settings are chosen as α=0.05,L=2,γ=150, for m=20 (left) and m=50 (right). Steps 1–3 of the simulation procedure are repeated for $N=10^{6}$ times, and the number of bootstrap samples is set to $B=10^{4}$. A rational approximation with three‐digit precision after the decimal point was selected.

Figure 4 highlights the differences in out‐of‐control performance between the unadjusted and adjusted methods. When interpreting the results in Figure 4, it is important to note that the figure includes unadjusted, approximate, and exact methods with fundamentally different in‐control guarantees, as the non‐exact methods do not maintain GICP adherence. In‐control and out‐of‐control behavior naturally exists on a balance, and therefore comparisons focusing solely on out‐of‐control behavior can be misleading. Specifically, alarms from non‐exact methods are often raised under looser adherence to the GICP condition as illustrated in Figures 1 and 3.

The exact adjusted limits result in the largest ${\text{AARL}}_{1}$ values. This effect is particularly pronounced when both m and $\lambda_{0}$ are small. However, for small values of both m and $\lambda_{0}$, non‐exact methods also show more extreme violations of the GICP condition, as illustrated in Figure 3. Compared to the exact method, the knowledge‐based exact method demonstrates better out‐of‐control performance for small $\lambda_{0}$ values, as condition (16) is satisfied for the considered parameters. Further, we verify that increasing the Phase I sample size m improves out‐of‐control performance.

Based on the simulation results, we recommend that practitioners employ the exact methods. However, since these attain the GICP condition conservatively (see Figure 3), they naturally entail reduced out‐of‐control performance. Accordingly, one may consider non‐exact methods in practical scenarios where exact adherence to the GICP condition is not required. Importantly though, the degree of adherence to the GICP condition among non‐exact methods can be highly variable and anti‐conservative, particularly when m and $\lambda_{0}$ are small. For larger values of these parameters—where approximate adherence is tolerable—non‐exact methods can be viable alternatives that yield modest improvements in out‐of‐control performance. Among the non‐exact methods, the bootstrap and approximate bootstrap approaches show greater anti‐conservatism and more variable adherence to the GICP condition than the quantile method. We therefore recommend the quantile method when exact GICP adherence is not required, and the achieved approximate adherence is acceptable.

## 6 Application: Chikungunya in International Travelers

We emphasize the practical utility of exact approaches through the application of the methodology to surveillance data of health records of returning international travelers, to retrospectively detect chikungunya outbreaks in the countries of travel. Given the limited availability of representative historical data, addressing estimation uncertainty is crucial for minimizing false alarms. False alarms in disease surveillance carry substantial costs, including the economic impact of negative travel advisories and reputational damage to the surveillance network due to a loss of confidence in its reliability (Nicola et al. 2020). The need for exact approaches becomes evident when stratifying the data at a country level, where disease counts become too small to reliably analyze using the existing methods described in Section 3.4.

Sentinel surveillance of international travelers has enabled GeoSentinel, a global surveillance and research network collaboration between the International Society of Travel Medicine and the US Centers for Disease Control and Prevention (CDC), to effectively identify multiple unrecognized outbreaks of public health importance (Hamer et al. 2020), complementing domestic surveillance efforts (Stoepker 2025). Its clinical sites are experienced in evaluating and treating patients with travel‐related infectious diseases and contribute anonymous clinician‐based surveillance data on ill persons who recently crossed an international border. Chikungunya virus, an alphavirus transmitted by female *Aedes* spp. mosquitoes, particularly *Aedes aegypti* and *Aedes albopictus*, poses a health risk to humans. Although chikungunya is usually not fatal, the associated morbidity may be significant and chronic; incapacitating arthralgias may persist for months to years following acute infection (Bierbrier et al. 2024).

Phase I, which is set as 2014–2016 in this case study, should contain non‐outbreak‐like behavior. However, during 2013–2015, a major chikungunya outbreak occurred in the Caribbean (Bierbrier et al. 2024). To maintain the integrity of the analysis, Caribbean countries are omitted from the analysis. To our knowledge, no substantial signs of further outbreaks during 2014–2016 have been documented in the GeoSentinel data (Bierbrier et al. 2024), suggesting that the risk of false negatives is low.

For this study, we used all confirmed and probable chikungunya diagnoses reported to the GeoSentinel database between 2014 and 2023, including data from countries (excluding the Caribbean) that recorded at least one chikungunya case during this period (86 countries, 934 total cases). The dates of the initial clinic visits served as timestamps. The data were aggregated on a per‐country, four‐week basis, mitigating the effects of clustered data entries, and day‐to‐day variations, such as fewer clinic visits on weekends. Cases from the 3‐year (Phase I) period 2014–2016 were used to estimate a baseline (m=39), and the 7‐year (Phase II) monitoring period was set as 2017–2023 (i.e., 91 4‐week periods).

For the parameters considered in this case study (m=39 and L=2), the condition (16), which indicates when the knowledge‐based exact method is more appropriate than the exact method, holds under the assumption that $\lambda_{0}\geq 0.004419$. This implies that the average number of 4‐week periods between in‐control cases must be at most 1/0.004419≈226, corresponding to a minimum baseline rate of one case every 17.4 years. We consider this assumption to be reasonable, as chikungunya is an endemic disease in many of the countries under consideration, and even in non‐endemic areas, secondary vector‐borne infections, for instance, due to airport infection are not uncommon (Ibañez‐Justicia et al. 2017; Alenou and Etang 2021). Consequently, we will adopt the knowledge‐based exact method for this case study.

Configuring the CUSUM design parameters as $L=2,{\text{ARL}}_{0}=150,\;\text{and}\;\alpha =0.05$, and employing the knowledge‐based exact method to all 86 countries, we anticipate a maximum of two false alarms per year under no‐outbreak conditions (Supporting Information Appendix G). This frequency is deemed manageable by travel epidemiologists.

To further investigate the potential outbreak, we referred to the five major review papers (Bierbrier et al. 2024; Khongwichit et al. 2021; Costa et al. 2023; Manzoor et al. 2022; Chinedu Eneh et al. 2023) and conducted a search on https://pubmed.ncbi.nlm.nih.gov/ by querying “Chikungunya + <Country> + <Year>” for the first 25 results. Additionally, reports of international public health authorities were included if found. In the absence of supporting articles or reports, we consider the alarm as a false alarm. Although we attempt to construct these definitions, it is important to note that no true ground truth definitions exist.

The application of knowledge‐based exact‐adjusted control limits effectively identified nine major chikungunya outbreaks in Bangladesh, Kenya, Thailand, Democratic Republic of the Congo, Sri Lanka, Maldives, Myanmar, Djibouti, and Paraguay, with a single false alarm in Equatorial Guinea (positive predictive value = 90%). The unadjusted method triggered more alarms (26), detecting more true positives (14) but also generating 12 false alarms. Its lower positive predictive value (54%) shows the practical necessity of incorporating estimation uncertainty. Further details of alarms raised by the knowledge‐based exact and unadjusted methods, including baseline rates, alarm dates, the number of cases and 4‐week periods used to trigger an alarm, and corresponding publications for true alarms, are presented in Supporting Information Appendix E. Analogous details for alarms triggered by the bootstrap, approximate bootstrap, quantile, and exact methods are provided in Supporting Information Appendix F.

Our case study shows the practical utility of our methodology. The resulting practical insights come with some limitations, which are not exclusive to the knowledge‐based exact method but extend to all the methods discussed within this paper. First, the underlying model is simplistic and does not incorporate potential spatial and temporal dependencies that may exist in our scenario. Although the precise impact of such autocorrelations on the GICP condition has not yet been quantified, they generally lead to increased model sensitivity (Lu and Reynolds 2001), potentially inflating the false alarm rate and compromising GICP adherence. Second, the model does not account for seasonal effects, such as those associated with rainfall patterns (Chinedu Eneh et al. 2023). Ignoring seasonality can result in periodic alarms during seasonal peaks, although no clear evidence of this was observed in our case study. Conversely, low‐magnitude outbreaks occurring during low‐season periods may be missed, as the in‐control guarantees during such times are overly conservative when seasonality is present. Nonetheless, as the absolute magnitude of these missed out‐of‐season outbreaks is small, the practical implications of this limitation may be minor. Third, the modeled incidence rates are assumed to arise from a population that is constant over time, assuming no substantial trends. This assumption may compromise the value of our inference, as this type of sentinel surveillance does not distinguish between an increase in case numbers due to an elevated chikungunya incidence rate or increased travel volume. Finally, within the GeoSentinel network, the time gap between the initial clinic visit and database reporting is non‐negligible. As a result, utilizing the date of the initial visit as a timestamp may raise alarms at an earlier date than they could have been raised during the data collection process.

## 7 Conclusion and Discussion

This paper addressed the problem of ensuring in‐control performance of the CUSUM chart in the context of small Poisson counts. We demonstrated that without accounting for estimation uncertainty, in‐control performance cannot be guaranteed. Existing bootstrap and approximate bootstrap methods were reviewed, and the quantile approach of Zhao and Driscoll (2016) was extended from c‐charts to the Poisson CUSUM chart. A heuristic for handling cases where all Phase I observations are zero was provided, as these methods fail without such an approach. Our findings showed that existing methods only approximately meet the GICP condition, with notable performance deviations when the mean count or Phase I sample size is small. Among these, the quantile approach showed slightly better GICP performance compared to the bootstrap and approximate bootstrap methods.

We then proposed two exact methods—the exact method and the knowledge‐based exact method—that guarantee the GICP condition and do not require heuristics when all Phase I observations are zero. Based on assumptions about the baseline rate, we provided guidance on selecting the appropriate method. The underlying theory of these methods extends beyond Poisson CUSUM charts and applies to any distribution where parameter ordering implies stochastic dominance, such as one‐parameter distributions in the exponential family. An illustration for the binomial distribution is included in Supporting Information Appendix C. Since in‐control and out‐of‐control performance naturally exist on a balance, the out‐of‐control performance of the exact approaches is diminished compared to bootstrap‐based methods. However, we have shown that if domain knowledge is available, in many practical scenarios, one can elevate the out‐of‐control performance of the chart through the usage of the knowledge‐based exact approach. This is further supported by the application to chikungunya outbreaks in international travelers, which confirms that sufficient detection power is retained for practical purposes. In applications where the GICP condition is not required to hold exactly, the quantile method may be a suitable alternative to the exact method. It should be noted, however, that the GICP adherence of the quantile method can be highly variable and anti‐conservative, particularly for small m and $\lambda_{0}$.

In this work, we considered Garwood upper confidence limits (Garwood 1936; Swift 2009). Alternative exact upper limits exist, which may be used in the proposed methodology instead: for example, Blaker (2000)'s confidence limits, which lower Garwood's upper limits while maintaining exactness. Such methods can improve out‐of‐control performance. However, the non‐nested structure of such intervals may yield conflicting results, leading to an alarm when the GICP condition is attained at level α but to no alarm at a higher level $\alpha^{\prime }>\alpha$. Such behavior may be undesirable in practice. Since we observe satisfactory detection power through the use of Garwood upper limits in Section 6, we recommend that the analyst to use Garwood upper limits in the proposed methodology.

To align with most existing literature, this paper is structured with a focus on the ARL as a performance metric. However, researchers may prefer the use of other statistics related to the run length due to its right‐skewed nature (Rizzo et al. 2020), such as the median run length, or quantiles of the run length. In such cases, our methodology can be adapted analogously, which we demonstrate in a supplementary analysis (Supporting Information Appendix D).

Recently, there has been a growing interest in parameter learning schemes, wherein Phase I parameters are updated with a delay (e.g., Capizzi and Masarotto 2020; Huberts et al. 2022; Zago and Capizzi 2024). Typically, in such scenarios, the initial Phase I sample size is relatively small. Therefore, employing exact methods instead of bootstrap‐based methods could strengthen the in‐control guarantees.

Our focus was on independent and identically distributed Poisson counts. However, analogous exact methodologies for other one‐parameter distributions can be formulated, provided an ordering on their parameters implies stochastic dominance (Section 4). Exploring the performance and practical applications of exact CUSUM charts for such distributions can offer insights across diverse domains. Moreover, it has been shown that bootstrap‐based methods can appropriately account for temporal correlations (Weiß et al. 2018). Extending these analyses, including considering spatial correlations, using exact methods, is an area that warrants further exploration in future research.

## Conflicts of Interest

The authors declare no conflicts of interest.

## Open Research Badges

This article has earned an Open Data badge for making publicly available the digitally‐shareable data necessary to reproduce the reported results. The data is available in the Supporting Information section.

This article has earned an open data badge “**Reproducible Research**” for making publicly available the code necessary to reproduce the reported results. “The results reported in this article could fully be reproduced.”

## Supporting information

Appendices, figures and tables referenced in Sections 3, 5, and 6 can be found online in the Supporting Information section. **Supporting File 1:** bimj70127‐sup‐0001‐DataCode.zip.

**Supporting File 2:** bimj70127‐sup‐0002‐Suppmat.pdf.

## Data Availability

Data and R codes used for simulation studies can be accessed at https://github.com/SHeidema/ExactPCUSUM. The data underlying the application were provided by GeoSentinel under licence/by permission. Data will be shared on request to the corresponding author with permission of GeoSentinel.
