# Supplementary material for: The Poisson CUSUM Chart for Monitoring Small Counts: Addressing the Estimation Uncertainty
Source: Biom J. 2026 Apr 5;68(2):e70127. doi: 10.1002/bimj.70127 (PMC13051258; doi:10.1002/bimj.70127)
Supplement: Supplementary file 2 — Supporting File 2: bimj70127‐sup‐0002‐Suppmat.pdf. [file BIMJ-68-e70127-s002.pdf]

Biometrical Journal **52** (2010) 61, zzz–zzz / DOI: 10.1002/bimj.200100000

The Poisson CUSUM chart for monitoring small counts: Addressing the estimation uncertainty - Supporting Information

Stan Heidema[*,1], **Ivo V. Stoepker** [1], **Ralph Huits** [2], and **Edwin R. van den Heuvel** [1]

[1] Department of Mathematics and Computer Science, Technische Universiteit Eindhoven, Groene Loper 5, 5612AZ, Eindhoven, The Netherlands

[2] Department of Infectious Tropical Diseases and Microbiology, IRCCS Sacro Cuore Don Calabria Hospital, Via Don A. Sempreboni, 5, 37024, Negrar di Valpolicella (VR), Italy

Received zzz, revised zzz, accepted zzz

This supporting information contains details on abbreviations, proofs and derivations, and details on the chikungunya case study.

*Corresponding author: e-mail: s.g.a.m.heidema@tue.nl

© 2010 WILEY-VCH Verlag GmbH & Co. KGaA, Weinheim

Web Appendix A: Abbreviations

This table summarizes the abbreviations used throughout the main document.

AARL	Average (Conditional) Average Run Length
ARL	Average Run Length
CARL	Conditional Average Run Length
CDC	Centers for Disease Control and Prevention
CQRL	Conditional Quantile of the Run Length
CUSUM	Cumulative Sum
GICP	Guaranteed In-Control Performance
ISTM	International Society of Travel Medicine
MRL	Median Run Length
QRL	Quantile of the Run Length
RSDARL	Relative Standard Deviation of (Conditional) Average Run Length
VARL	Variance of (Conditional) Average Run Length

Web Appendix B: Proof of Lemma 3.1

Here, we prove Lemma 3.1, demonstrating that stochastic domination between random variables leads to monotonic control limits. For Poisson distributions P_λ and $P_{\lambda'}$, stochastic domination is equivalent to the ordering $\lambda \leq \lambda'$.

Lemma 3.1 *Let $Y_1, Y_2, \ldots \sim F$ and $Y_1', Y_2', \ldots \sim F'$ be independent random variables such that for all $t \in \mathbb{N}$, Y_t is stochastically dominated by Y_t'. Then $h(F,k) \leq h(F',k)$ for all $k \geq 0$.*

Proof. Let $k \geq 0$ be arbitrary, and let $\mathbf{Y} = (Y_1, Y_2, \ldots) \sim F$, and let $\mathbf{Y}' = (Y_1', Y_2', \ldots) \sim F'$ be independent random variables such that for all $t \in \mathbb{N}$, Y_t is stochastically dominated by Y_t'. Denote with $C_t(k)$ and $C_t'(k)$ the control charts induced by these variables, each with reference value k.

It will be sufficient to show that the sequence of CUSUM statistics $C_1(k), \ldots, C_t(k)$ is stochastically dominated by $C_1'(k), \ldots, C_t'(k)$, i.e.

$$\mathbb{P}[C_1'(k) \leq h, \ldots, C_t'(k) \leq h] \leq \mathbb{P}\left[C_1(k) \leq h, \ldots, C_t(k) \leq h\right] \ . \tag{1}$$

for all $t \in \mathbb{N}$ and $h \geq 0$. To see why this suffices, note that the stochastic domination above implies (reversed) stochastic domination of the run length distributions as:

$$\begin{aligned} \mathbb{P}_{\mathbf{Y}'\sim F'}\left[\tau(k,h) \geq t\right] &= \mathbb{P}[C_1'(k) \leq h, \ldots, C_t'(k) \leq h] \\ &\overset{(1)}{\leq} \mathbb{P}\left[C_1(k) \leq h, \ldots, C_t(k) \leq h\right] \\ &= \mathbb{P}_{\mathbf{Y}\sim F}\left[\tau(k,h) \geq t\right] \ . \end{aligned} \tag{2}$$

for all $t \in \mathbb{N}$ and $h \geq 0$. Since $\tau(k,h)$ is nonnegative the above implies an ordering of their moments:

$$\mathbb{E}_{\mathbf{Y}'\sim F'}\left[\tau(k,h)\right] \leq \mathbb{E}_{\mathbf{Y}\sim F}\left[\tau(k,h)\right] \text{ for all } h \geq 0 \ . \tag{3}$$

© 2010 WILEY-VCH Verlag GmbH & Co. KGaA, Weinheim

Finally, the above implies the statement of the lemma as

$$h(F,k) = \inf\{h \geq 0 : \mathbb{E}_{\mathbf{Y}\sim F}[\tau(k,h)] \geq \gamma\}$$
$$\leq \inf\{h \geq 0 : \mathbb{E}_{\mathbf{Y}'\sim F'}[\tau(k,h)] \geq \gamma\} = h(F',k)\,. \tag{4}$$

What remains to be proven is the stochastic domination in (1). We prove this by induction. Note that for all t we have that Y_t is stochastically dominated by Y'_t. Therefore, by Strassen's Theorem (Strassen [1965]), for all t there exists a coupling $(\tilde{Y}_t, \tilde{Y}'_t)$ of Y_t and Y'_t such that

$$\mathbb{P}\left[\tilde{Y}_t \leq \tilde{Y}'_t\right] = 1\,. \tag{5}$$

Fix a control limit $h \geq 0$. Then for the base case, as $C_0(k) = 0$,

$$\begin{aligned}
\mathbb{P}[C'_1(k) \leq h] &= \mathbb{P}\left[\max\left(0, Y'_1 - k\right) \leq h\right] \\
&= \mathbb{P}\left[\max\left(0, \tilde{Y}'_1 - k\right) \leq h\right] \\
&= \mathbb{P}\left[\max\left(0, \tilde{Y}'_1 - k\right) \leq h \;\middle|\; \tilde{Y}_1 \leq \tilde{Y}'_1\right] \\
&\leq \mathbb{P}\left[\max\left(0, \tilde{Y}_1 - k\right) \leq h \;\middle|\; \tilde{Y}_1 \leq \tilde{Y}'_1\right] \\
&= \mathbb{P}\left[C_1(k) \leq h \;\middle|\; \tilde{Y}_1 \leq \tilde{Y}'_1\right] = \mathbb{P}[C_1(k) \leq h]\,.
\end{aligned} \tag{6}$$

Now, as the inductive hypothesis, we assume that the stochastic dominance condition holds for an arbitrary $t \in \mathbb{N}$:

$$\mathbb{P}[C'_1(k) \leq h, \ldots, C'_t(k) \leq h] \leq \mathbb{P}[C_1(k) \leq h, \ldots, C_t(k) \leq h]\,. \tag{7}$$

For $t+1$ we note that

$$\begin{aligned}
&\mathbb{P}\big[C'_{t+1}(k) \leq h \big| C'_t(k) \leq h\big] \\
&= \int_0^h \mathbb{P}\big[C'_{t+1}(k) \leq h \big| C'_t(k) = x\big]\,dx \\
&= \int_0^h \mathbb{P}\big[\max(0, x + Y'_{t+1} - k) \leq h \big| C'_t(k) = x\big]\,dx \\
&= \int_0^h \mathbb{P}\big[\max(0, x + \tilde{Y}'_{t+1} - k) \leq h \big| \tilde{Y}_{t+1} \leq \tilde{Y}'_{t+1}, C'_t(k) = x\big]\,dx \\
&\leq \int_0^h \mathbb{P}\big[\max(0, x + \tilde{Y}_{t+1} - k) \leq h \big| \tilde{Y}_{t+1} \leq \tilde{Y}'_{t+1}, C'_t(k) = x\big]\,dx \\
&= \int_0^h \mathbb{P}\big[C_{t+1}(k) \leq h \big| C_t(k) = x\big]\,dx \\
&= \mathbb{P}\big[C_{t+1}(k) \leq h \big| C_t(k) \leq h\big].
\end{aligned} \tag{8}$$

© 2010 WILEY-VCH Verlag GmbH & Co. KGaA, Weinheim

Therefore,

$$\begin{aligned}
&\mathbb{P}\big[C_1'(k) \le h, \dots, C_{t+1}'(k) \le h\big] \\
&\quad = \mathbb{P}\big[C_{t+1}'(k) \le h \;\big|\; C_1'(k) \le h, \dots, C_t'(k) \le h\big] \cdot \mathbb{P}\big[C_1'(k) \le h, \dots, C_t'(k) \le h\big] \\
&\quad = \mathbb{P}\big[C_{t+1}'(k) \le h \;\big|\; C_t'(k) \le h\big] \cdot \mathbb{P}\big[C_1'(k) \le h, \dots, C_t'(k) \le h\big] \\
&\quad \overset{(7)}{\le} \mathbb{P}\big[C_{t+1}(k) \le h \;\big|\; C_t(k) \le h\big] \cdot \mathbb{P}\big[C_1(k) \le h, \dots, C_t(k) \le h\big] \\
&\quad \overset{(8)}{\le} \mathbb{P}\big[C_{t+1}(k) \le h \;\big|\; C_t(k) \le h\big] \cdot \mathbb{P}\big[C_1'(k) \le h, \dots, C_t'(k) \le h\big] \\
&\quad = \mathbb{P}\big[C_1(k) \le h, \dots, C_{t+1}(k) \le h\big],
\end{aligned} \tag{9}$$

concluding the inductive argument and implying (1), thereby concluding the proof. □

Web Appendix C: Illustration of the Exact Method for the Binomial Distribution

To illustrate that the exact method extends to one-parameter distributions in the exponential family, we also conducted simulations under the binomial distribution with a known population size $N \in \mathbb{N}$ and an unknown proportion $q \in [0,1]$. Specifically, let $Y_1, Y_2, \dots \overset{\text{i.i.d.}}{\sim} \text{Bin}(N, q)$, and estimate q based on a finite sample $\mathbf{X} = (X_1, \dots, X_m) \overset{\text{i.i.d.}}{\sim} \text{Bin}(N, q_0)$ as

$$\hat{q}_0 = \frac{1}{Nm} \sum_{i=1}^{m} X_i. \tag{10}$$

An appropriate $(1-\alpha) \cdot 100\%$ upper limit for q can be constructed using the Clopper–Pearson interval (Clopper and Pearson [1934]):

$$U_\alpha(\mathbf{X}) = \begin{cases} 1, & \text{if } \sum_{i=1}^{m} X_i = Nm, \\ B^{-1}(1-\alpha; \sum_{i=1}^{m} X_i + 1,\, N - \sum_{i=1}^{m} X_i), & \text{if } \sum_{i=1}^{m} X_i < Nm, \end{cases} \tag{11}$$

where B^{-1} denotes the quantile function of the Beta distribution. Consistent with the Poisson chart, we set the out-of-control parameter q_1 as an additive shift of L standard deviations, while accounting for the constraint $q_1 \le 1$. Specifically, we define

$$q_1 = \min\left(q_0 + L\sqrt{\frac{q_0(1-q_0)}{N}}, 1\right), \tag{12}$$

and set the reference value to

$$k_{q_0} = -\frac{N \log\left(\frac{1-q_1}{1-q_0}\right)}{\log\left(\frac{q_1(1-q_0)}{q_0(1-q_1)}\right)}, \tag{13}$$

aligning the chart with a scaled version of the Wald sequential probability ratio test statistic for the binomial distribution (Wald [1945]). Analogous to Theorem 4.1, the exact control limit for binomial data can be constructed as $h(P_{U_\alpha}, k_{\hat{q}_0})$.

As the `spc` package does not provide efficient Markov chain procedures for the binomial distribution, we opt for Monte Carlo simulation for this scenario. Figure 1 shows the resulting control limits. Figure 2 illustrates the adherence to the GICP condition.

© 2010 WILEY-VCH Verlag GmbH & Co. KGaA, Weinheim

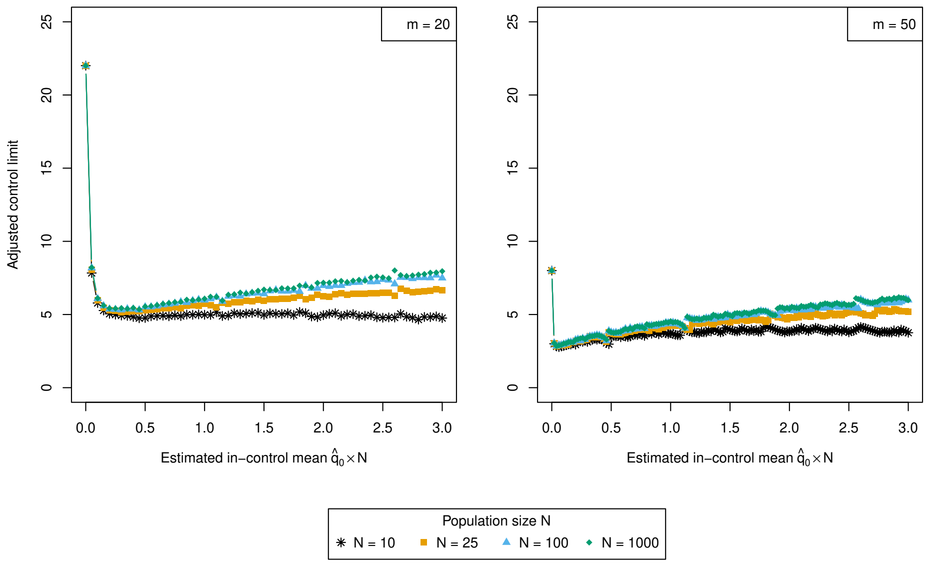

Figure 1 Various control limits with respect to estimated in-control mean $\hat{q}_0 N$ as produced by the exact method for binomial data, employing Clopper-Pearson upper limits, for $N = 10, 25, 100, 1000$. Parameter settings are chosen as $\alpha = 0.05, L = 2, \gamma = 150$, for $m = 20$ (left) and $m = 50$ (right).

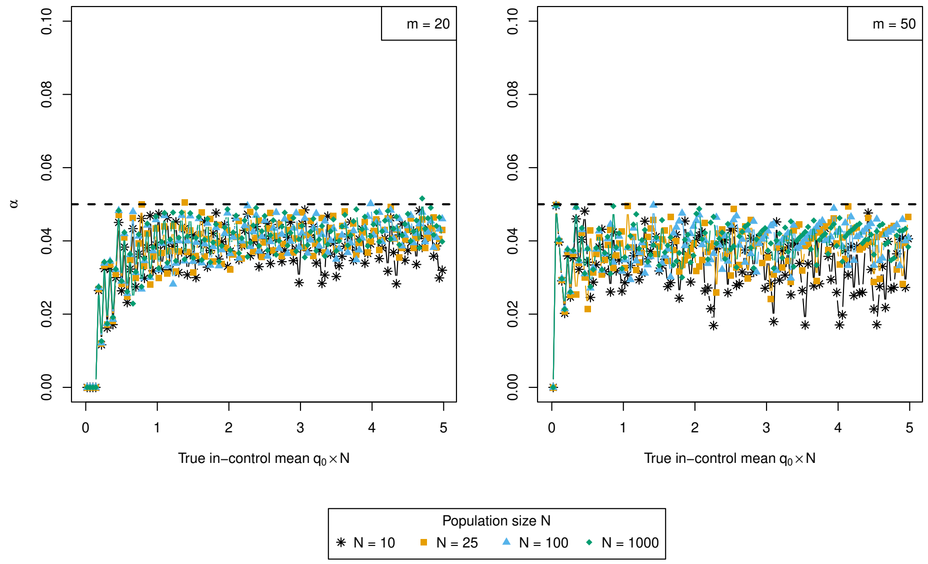

Figure 2 Guaranteed in-control performance with respect to the true in control mean $q_0 N$ when adjusting control limits according to the exact method for binomial data, employing Clopper-Pearson upper limits, with nominal value $\alpha = 0.05$. Remaining parameters are $L = 2, \gamma = 150$, for $m = 20$ (left) and $m = 50$ (right). Steps 1-3 of the simulation procedure are repeated for $N = 10^6$ times, leading to estimates of the attained α.

© 2010 WILEY-VCH Verlag GmbH & Co. KGaA, Weinheim

Web Appendix D: Adapting the Exact Method for Alternative Run Length Metrics

While the main text focuses on the average run length (ARL) as the primary performance metric, the ARL is known to be highly right-skewed, which can limit its interpretability in practice. Alternative summaries of the run length distribution, such as the median run length (MRL) or conditional quantiles, are sometimes preferred in applied settings (Rizzo et al. [2020]). In this section, we show how to construct exact control limits that satisfy an analogous GICP condition for these alternative metrics.

Analogous to the ARL, we define the u-th quantile of the run length as

$$\mathrm{QRL}_u(P, k, h) = \min\left\{n \in \mathbb{N} : \mathbb{P}_{\mathbf{Y}\sim P}\left[\tau(k, h) \leq n\right] \geq u\right\} \tag{14}$$

We consider control limits which are chosen to ensure that the procedure attains a pre-specified nominal in-control u-th quantile of the run length of at least γ, where $u \in (0, 1)$ is a fixed predetermined quantile level of choice. Specifically,

$$h_u(P, k) = \inf\{h \geq 0 : \mathrm{QRL}_u(P, k, h) \geq \gamma\} \tag{15}$$

denotes the smallest control limit guaranteeing that the u-th quantile of the run length distribution is at least γ, with an in-control Phase II distribution P, and the reference value given by k. Analogous to Lemma 3.1, we formulate Lemma D.1:

Lemma D.1: *Let $Y_1, Y_2, \ldots \sim F$ and $Y_1', Y_2', \ldots \sim F'$ be independent univariate random variables such that for all $t \in \mathbb{N}$, Y_t is stochastically dominated by Y_t', and let $u \in (0, 1)$. Then $h_u(F, k) \leq h_u(F', k)$ for all $k \geq 0$.*

Proof. The argument parallels that of Lemma 3.1, except for steps (3) and (4). Note that stochastic domination of the run length distributions, as stated in (2), implies

$$\mathrm{QRL}_u(F', k, h) \leq \mathrm{QRL}_u(F, k, k) \tag{16}$$

Consequently,

$$\begin{aligned} h_u(F, k) &= \inf\{h \geq 0 : \mathrm{QRL}_u(F, k, h) \geq \gamma\} \\ &\leq \inf\{h \geq 0 : \mathrm{QRL}_u(F', k, h) \geq \gamma\} = h_u(F', k) \end{aligned} \tag{17}$$

□

As before, for clarity and concreteness we focus on the Poisson distribution. For a choice of the reference value $k(\mathbf{X})$, the control limit $h(\mathbf{X})$ and a fixed quantile level $u \in (0, 1)$, the conditional quantile of the run length is defined as

$$\mathrm{CQRL}_u\Big(k(\mathbf{X}), h(\mathbf{X}), \mathbf{X}, \lambda_0\Big) = \min\left\{n \in \mathbb{N} : \mathbb{P}_{\mathbf{Y}\sim P_{\lambda_0}}\left[\tau\big(k(\mathbf{X}), h(\mathbf{X})\big) \leq n \,\middle|\, \mathbf{X}\right] \geq u\right\}. \tag{18}$$

Adapting the uniform GICP condition for conditional quantiles of the run length distribution, we require

$$\inf_{\lambda_0 > 0} \mathbb{P}_{\mathbf{X}\sim P_{\lambda_0}}\left[\mathrm{CQRL}_u\Big(k(\mathbf{X}), h(\mathbf{X}), \mathbf{X}, \lambda_0\Big) \geq \gamma\right] \geq 1 - \alpha. \tag{19}$$

In analogy with Theorem 4.1, we state Theorem D.2 as follows:

© 2010 WILEY-VCH Verlag GmbH & Co. KGaA, Weinheim

Theorem D.2: *Let U_α be an $(1-\alpha)\cdot 100\%$ upper limit such that*

$$\inf_{\lambda_0 \geq 0} \mathbb{P}[\lambda_0 \leq U_\alpha] = 1 - \alpha. \tag{20}$$

Then for any Phase I data dependent reference value $k(\mathbf{X}) \geq 0$, and fixed quantile level $u \in (0,1)$, the CUSUM chart with control limit $h_u(P_{U_\alpha}, k(\mathbf{X}))$ uniformly guarantees the GICP condition (19) at level α.

Proof. The proof follows the same reasoning as Theorem 4.1. Assume that (20) holds, and let $k \geq 0$ be fixed. Then, by Lemma D.1 we have with at least $1-\alpha$ probability that

$$h_u(P_{\lambda_0}, k) \leq h_u(P_{U_\alpha}, k) \text{ for all } \lambda_0 \geq 0. \tag{21}$$

Moreover, under a sequence of Phase II observations distributed according to P_{λ_0}, if control limits satisfy $h \leq h'$, then the corresponding run lengths are ordered almost surely, i.e., $\mathbb{P}_{\mathbf{Y}\sim P_{\lambda_0}}[\tau(k,h) \leq \tau(k,h')] = 1$. This implies that whenever $h \leq h'$:

$$\text{QRL}_u(P_{\lambda_0}, k, h) \leq \text{QRL}_u(P_{\lambda_0}, k, h') \tag{22}$$

Combining (21) and (22), it follows that, with at least probability $1-\alpha$, for all $\lambda_0 \geq 0$:

$$\begin{aligned}\gamma &\leq \min\Big\{n \in \mathbb{N} : \mathbb{P}_{\mathbf{Y}\sim P_{\lambda_0}}\Big[\tau\Big(k(\mathbf{X}), h_u\big(P_{\lambda_0}, k(\mathbf{X})\big)\Big) \leq n \,\Big|\, \mathbf{X}\Big] \geq u\Big\} \\ &\leq \min\Big\{n \in \mathbb{N} : \mathbb{P}_{\mathbf{Y}\sim P_{\lambda_0}}\Big[\tau\Big(k(\mathbf{X}), h_u\big(P_{U_\alpha}, k(\mathbf{X})\big)\Big) \leq n \,\Big|\, \mathbf{X}\Big] \geq u\Big\} \\ &= \text{CQRL}_u\Big(k(\mathbf{X}), h_u\big(P_{U_\alpha}, k(\mathbf{X})\big), \mathbf{X}, \lambda_0\Big).\end{aligned} \tag{23}$$

□

If we construct upper limits U_α according to Garwood [1936]'s method, then $h_u(P_{U_\alpha}, k_{\hat{\lambda}_0^{\text{MLE}}})$ is the corresponding exact control limit, and the knowledge-based exact control limit for this scenario can be computed as $h_u(P_{U_\alpha}, k_{\hat{\lambda}_0})$. As the `spc` package does not provide efficient Markov chain procedures for these alternative run length metrics, we opt for Monte Carlo simulation. Figure 3 shows the resulting exact control limits for selected quantiles, and Figure 4 illustrates the adherence to the GICP condition (19). The setting where $u = 0.5$ corresponds to a scenario where the median run length is required to guarantee the in-control performance condition (19).

Web Appendix E: Application: Main Figures and Tables

This Section provides additional details on the results referred to in Section 6 of the main document. Figure 5 presents the cases and corresponding CUSUM statistics of the ten countries of exposure where the application of knowledge-based exact-adjusted control limits triggered an alarm. Table 1 provides an overview of alarms generated by the knowledge-based exact method. It includes the countries of exposure, alarm dates, the baseline rate during the three-year baseline period, as well as the alarm rate and number of 4-week periods that were used to produce an alarm. Additionally, the table shows the corresponding publication and the date at which the unadjusted method produced an alarm. Tables 2 and 3 show similar overviews for the alarms generated by the unadjusted method, but not by the knowledge-based exact method, showing alarms with and without corresponding publications respectively. The numbers in round brackets (1) and (2) following 'Indonesia' indicate whether we refer to the first or second alarm in Indonesia. Analogous tables for the bootstrap, approximate bootstrap, quantile, and exact methods are presented in Web Appendix D.

© 2010 WILEY-VCH Verlag GmbH & Co. KGaA, Weinheim

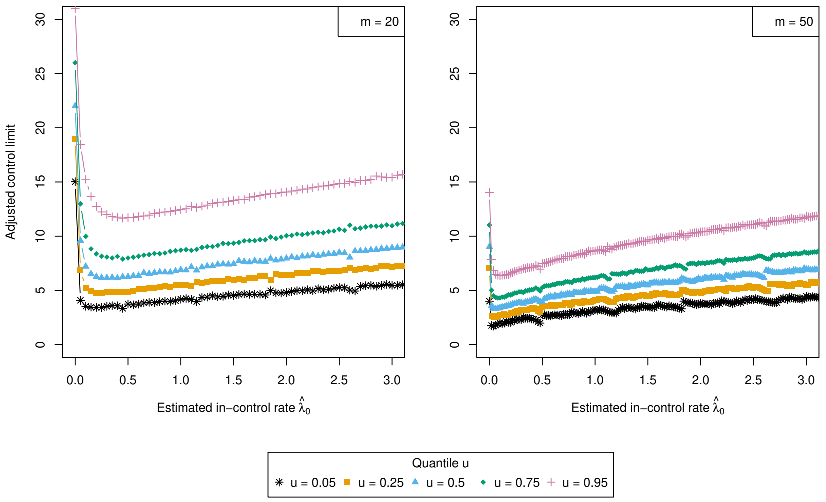

Figure 3 Various control limits with respect to estimated in-control rates $\hat{\lambda}_0$ as produced by the exact procedure where the primary performance metric is the conditional quantile of the run length, given five different levels: $u = 0.05, 0.25, 0.5, 0.75, 0.95$. The setting with $u = 0.5$ corresponds to a scenario where the median run length is the primary performance metric. Further parameter settings are chosen as $\alpha = 0.05, L = 2, \gamma = 150$, for $m = 20$ (left) and $m = 50$ (right).

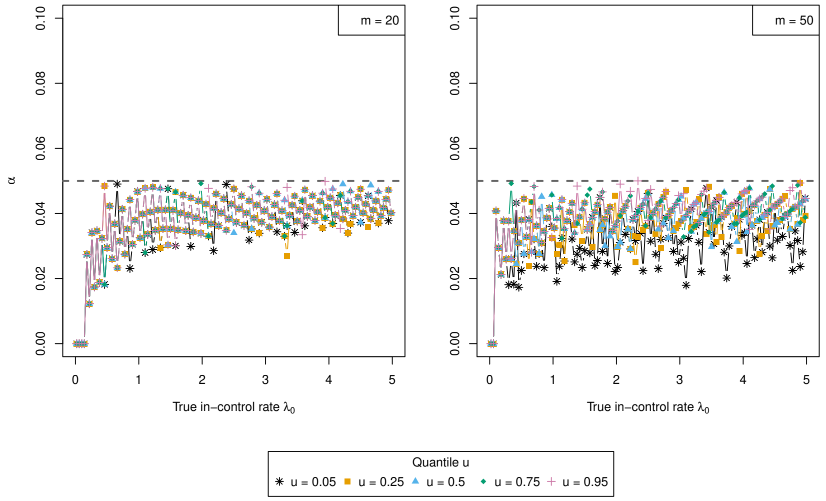

Figure 4 Guaranteed in-control performance relative to the true in-control rate λ_0, achieved by adjusting control limits using the exact procedure. The primary performance metric is the conditional quantile of the run length at five levels: $u = 0.05, 0.25, 0.50, 0.75, 0.95$. The nominal significance level is $\alpha = 0.05$. Remaining parameters: $L = 2$, $\gamma = 150$, with $m = 20$ (left) and $m = 50$ (right). Steps 1-3 of the simulation procedure are repeated $N = 10^6$ times, leading to estimates of the attained α.

© 2010 WILEY-VCH Verlag GmbH & Co. KGaA, Weinheim

Table 1 Alarms as generated by the knowledge-based exact-adjusted control limits and the unadjusted control limits. Note that alarm cases per 4-week period and the number of 4-week periods used for an alarm are calculated for the knowledge-based exact method, and generally differ for the unadjusted method.

Country of exposure	Date of alarm (knowledge-based exact method)	Baseline cases per 4-week period	Alarm cases per 4-week period	Months used for alarm	Publication	Date of alarm (unadjusted method)
Bangladesh	2017-08-09	0	0.6667	6	Amin et al. [2022]	2017-07-12
Kenya	2018-07-11	0.0513	0.7143	7	Eyase et al. [2020]	2018-02-21
Thailand	2019-01-23	0.1538	7	1	Javelle et al. [2019]	2019-01-23
DR Congo	2019-03-20	0	0.8	5	De Weggheleire et al. [2021]	2019-03-20
Sri Lanka	2019-04-17	0	0.2381	21	Abeygoonawardena et al. [2023]	2017-12-27
Maldives	2019-08-07	0.0256	1	6	Dudouet et al. [2020]	2019-06-12
Myanmar	2019-09-04	0.0513	8	1	Díaz-Menéndez et al. [2020]	2019-09-04
Equatorial Guinea	2019-10-02	0	0.3125	16	No	2018-09-05
Djibouti	2020-01-22	0	2	2	Geleta et al. [2020]	2019-12-25
Paraguay	2023-02-15	0.0256	12	1	Torales et al. [2023] + alert	2023-02-15

Table 2 Alarms with publications as generated by the unadjusted control limits, but not by the knowledge-based exact-adjusted limits.

Country of exposure	Date of alarm	Baseline cases per 4-week period	Alarm cases per 4-week period	Number of 4-week periods	Publication
Congo	2019-04-17	0.0256	2	1	Vairo et al. [2020]
Cameroon	2022-10-26	0.0256	0.25	8	Djeunang Dongho et al. [2022]
Indonesia (2)	2022-07-06	0.2564	2	2	Mayer et al. [2023]
Argentina	2023-07-05	0	1	2	PAHO and WHO articles

© 2010 WILEY-VCH Verlag GmbH & Co. KGaA, Weinheim

Table 3 Alarms without publications as generated by the unadjusted control limits, but not by the knowledge-based exact-adjusted limits.

Country of exposure	Date of alarm	Baseline cases per 4-week period	Alarm cases per 4-week period	Number of 4-week periods
Bolivia	2017-06-14	0.1282	1	3
Philippines	2017-10-04	0.1538	1.5	2
Indonesia (1)	2018-07-11	0.2564	4	1
Eritrea	2019-01-23	0	0.5	4
Guatemala	2019-10-30	0.0769	2	1
Senegal	2019-11-27	0.0256	1	2
Benin	2020-09-30	0.0256	0.6667	3
Mozambique	2021-04-14	0	2	1
Pakistan	2022-04-13	0	0.3333	6
Papua New Guinea	2022-11-23	0	0.25	8
Guinea	2023-08-30	0.0256	0.3333	6
India	2023-11-22	1.1538	3.3333	3

© 2010 WILEY-VCH Verlag GmbH & Co. KGaA, Weinheim

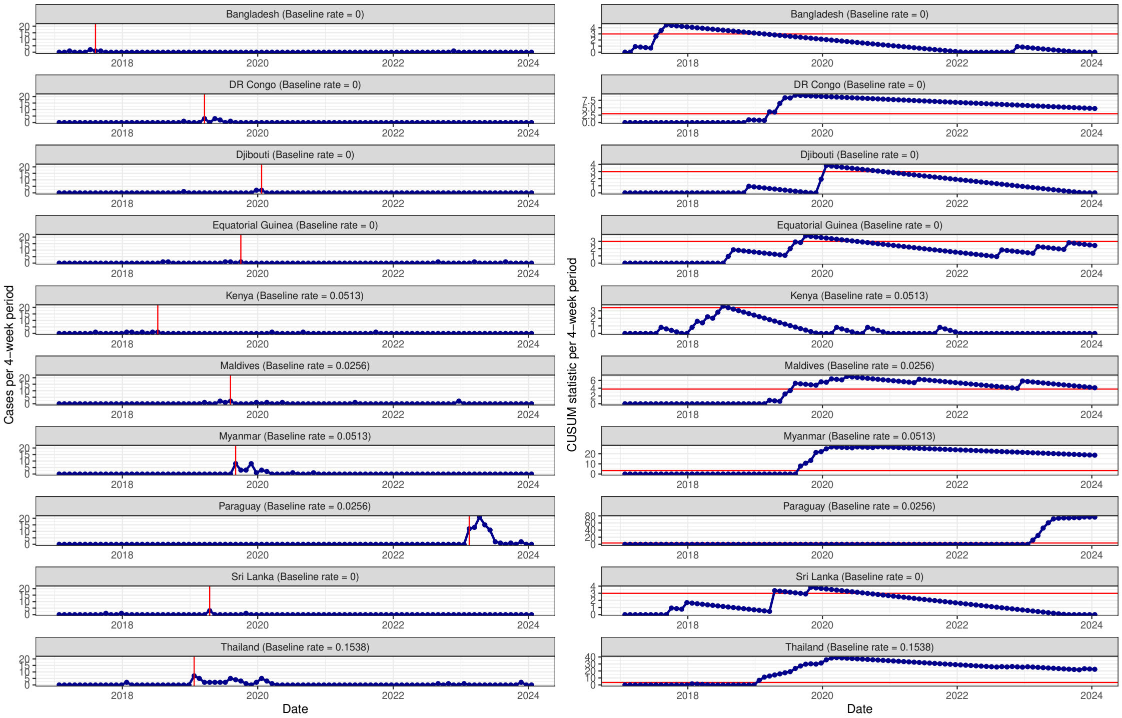

Figure 5 Cases per 4-week period (left) and the corresponding CUSUM statistic (right) between 2017-2023 for the ten countries for which the CUSUM chart exceeded the knowledge-based exact-adjusted control limits.

Web Appendix F: Application: Alarms raised by bootstrap, approximate bootstrap, and exact methods

For completeness, this Section presents alarms raised by the bootstrap, approximate bootstrap, quantile, and exact methods, which were not explicitly discussed in the Section 6 of the main document. Tables 4, 5, 6 and 7 provide overviews of alarms generated by the bootstrap, approximate bootstrap, quantile and exact methods respectively. The numbers in round brackets (1) and (2) following 'Indonesia' indicate whether we refer to the first or second alarm in Indonesia.

© 2010 WILEY-VCH Verlag GmbH & Co. KGaA, Weinheim

Table 4 Alarms as generated by the bootstrap-adjusted control limits

Country of exposure	Date of alarm	Baseline cases per 4-week period	Alarm cases per 4-week period	Months used for alarm	Publication
Bangladesh	2017-07-12	0	0.6	5	Amin et al. [2022]
Sri Lanka	2017-12-27	0	0.5	4	Abeygoonawardena et al. [2023]
Kenya	2018-04-18	0.0513	0.75	4	Eyase et al. [2020]
Equatorial Guinea	2018-09-05	0	1	2	No
Eritrea	2019-01-23	0	0.5	4	No
Thailand	2019-01-23	0.1538	7	1	Javelle et al. [2019]
DR Congo	2019-03-20	0	0.8	5	De Weggheleire et al. [2021]
Congo	2019-04-17	0.0256	2	1	Vairo et al. [2020]
Maldives	2019-06-12	0.0256	0.75	4	Dudouet et al. [2020]
Myanmar	2019-09-04	0.0513	8	1	Díaz-Menéndez et al. [2020]
Senegal	2019-11-27	0.0256	1	2	No
Djibouti	2019-12-25	0	2	1	Geleta et al. [2020]
Benin	2020-09-30	0.0256	0.6667	3	No
Mozambique	2021-04-14	0	2	1	No
Pakistan	2022-04-13	0	0.3333	6	No
Cameroon	2022-10-26	0.0256	0.25	8	Djeunang Dongho et al. [2022]
Papua New Guinea	2022-11-23	0	0.25	8	No
Paraguay	2023-02-15	0.0256	12	1	Torales et al. [2023] + alert
Argentina	2023-07-05	0	1	2	PAHO and WHO articles
Guinea	2023-08-30	0.0256	0.3333	6	No

© 2010 WILEY-VCH Verlag GmbH & Co. KGaA, Weinheim

Table 5 Alarms as generated by the approximate bootstrap-adjusted control limits.

Country of exposure	Date of alarm	Baseline cases per 4-week period	Alarm cases per 4-week period	Months used for alarm	Publication
Bangladesh	2017-07-12	0	0.6	5	Amin et al. [2022]
Sri Lanka	2017-12-27	0	0.5	4	Abeygoonawardena et al. [2023]
Kenya	2018-04-18	0.0513	0.75	4	Eyase et al. [2020]
Indonesia (1)	2018-07-11	0.2564	4	1	No
Equatorial Guinea	2018-09-05	0	1	2	No
Eritrea	2019-01-23	0	0.5	4	No
Thailand	2019-01-23	0.1538	7	1	Javelle et al. [2019]
DR Congo	2019-03-20	0	0.8	5	De Weggheleire et al. [2021]
Congo	2019-04-17	0.0256	2	1	Vairo et al. [2020]
Maldives	2019-06-12	0.0256	0.75	4	Dudouet et al. [2020]
Myanmar	2019-09-04	0.0513	8	1	Díaz-Menéndez et al. [2020]
Senegal	2019-11-27	0.0256	1	2	No
Djibouti	2019-12-25	0	2	1	Geleta et al. [2020]
Benin	2020-09-30	0.0256	0.6667	3	No
Mozambique	2021-04-14	0	2	1	No
Pakistan	2022-04-13	0	0.3333	6	No
Cameroon	2022-10-26	0.0256	0.25	8	Djeunang Dongho et al. [2022]
Papua New Guinea	2022-11-23	0	0.25	8	No
Paraguay	2023-02-15	0.0256	12	1	Torales et al. [2023] + alert
Argentina	2023-07-05	0	1	2	PAHO and WHO articles
Guinea	2023-08-30	0.0256	0.3333	6	No

© 2010 WILEY-VCH Verlag GmbH & Co. KGaA, Weinheim

Table 6 Alarms as generated by the quantile-adjusted control limits.

Country of exposure	Date of alarm	Baseline cases per 4-week period	Alarm cases per 4-week period	Months used for alarm	Publication
Bangladesh	2017-07-12	0	0.6	5	Amin et al. [2022]
Kenya	2018-06-13	0.0513	0.6667	6	Eyase et al. [2020]
Thailand	2019-01-23	0.1538	7	1	Javelle et al. [2019]
DR Congo	2019-03-20	0	0.8	5	De Wegheleire et al. [2021]
Sri Lanka	2019-04-17	0	0.2381	21	Abeygoonawardena et al. [2023]
Congo	2019-05-15	0.0256	1.5000	2	Vairo et al. [2020]
Maldives	2019-06-12	0.0256	0.75	4	Dudouet et al. [2020]
Equatorial Guinea	2019-07-10	0	0.2308	13	No
Myanmar	2019-09-04	0.0513	8	1	Díaz-Menéndez et al. [2020]
Eritrea	2019-10-30	0	0.2143	14	No
Djibouti	2019-12-25	0	2	1	Geleta et al. [2020]
Mozambique	2021-04-14	0	2	1	No
Paraguay	2023-02-15	0.0256	12	1	Torales et al. [2023] + alert

Table 7 Alarms as generated by the exact-adjusted control limits.

Country of exposure	Date of alarm (knowledge-based exact method)	Baseline cases per 4-week period	Alarm cases per 4-week period	Months used for alarm	Publication
Kenya	2018-07-11	0.0513	0.7143	7	Eyase et al. [2020]
Thailand	2019-01-23	0.1538	7	1	Javelle et al. [2019]
Maldives	2019-08-07	0.0256	1	6	Dudouet et al. [2020]
Myanmar	2019-09-04	0.0513	8	1	Díaz-Menéndez et al. [2020]
Paraguay	2023-02-15	0.0256	12	1	Torales et al. [2023] + alert

© 2010 WILEY-VCH Verlag GmbH & Co. KGaA, Weinheim

Web Appendix G: Estimation of false alarm rate in Application

In Section 6, the knowledge-based exact-adjusted control limit is calculated such that

$$\inf_{\lambda_0>0} \mathbb{P}_{\mathbf{X}\sim P_{\lambda_0}} \left[\text{CARL}\left(k_{\hat{\lambda}_0}, h(P_{U_\alpha}, k_{\hat{\lambda}_0}), \mathbf{X}, \lambda_0\right) \geq 150\right] \geq 0.95 \tag{24}$$

To estimate a false alarm rate, Figure 6 shows the simulated probability that the conditional run length is less than the total monitoring period of 91 4-week periods. We observe that with 95% confidence:

$$\mathbb{P}_{\mathbf{Y}\sim P_{\lambda_0}} \left[\tau\left(k_{\hat{\lambda}_0}, h(P_{U_\alpha}, k_{\hat{\lambda}_0}\right) \leq 91 \,\middle|\, \mathbf{X}\right] \leq 0.1455 \text{ for all } \lambda_0 \in (0, 3]. \tag{25}$$

Therefore, assuming that $\lambda_0 \leq 3$ for all countries, the expected number of false alarms during the entire monitoring period is at most this probability times the number of monitored countries, i.e. $0.1455 \cdot 86 = 12.51$.

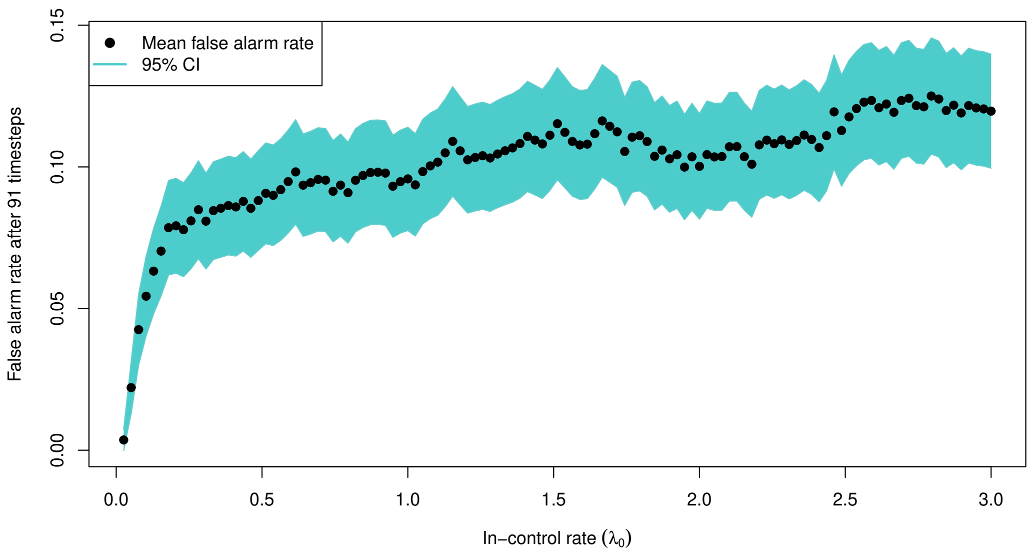

Figure 6 Mean false alarm rate with 95% confidence intervals after 91 timesteps, given the configurations of the case study.

© 2010 WILEY-VCH Verlag GmbH & Co. KGaA, Weinheim

References

H. Abeygoonawardena, N. Wijesinghe, V. Navaratne, A. Balasuriya, T. Nguyen, M. Moi, et al. Serological evidence of Zika virus circulation with dengue and chikungunya infections in Sri Lanka from 2017. *Journal of Global Infectious Diseases*, 15(3):113–120, 7 2023. ISSN 09748245. doi: 10.4103/jgid.jgid{_}195{_}22.

M. R. Amin, M. J. Hasan, M. A. S. Khan, M. A. Rafi, R. Islam, T. Shams, et al. Chikungunya outbreak in Bangladesh (2017): sociodemographic and clinical characteristics of patients from three hotspots. *Tropical Medicine and Health*, 50(1), 12 2022. ISSN 13494147. doi: 10.1186/s41182-022-00399-3.

C. J. Clopper and E. S. Pearson. The use of confidence or fiducial limits illustrated in the case of the binomial. *Biometrika*, 26:404–413, 1934. ISSN 0006-3444. doi: 10.1093/biomet/26.4.404.

A. De Weggheleire, A. Nkuba-Ndaye, P. Mbala-Kingebeni, J. Mariën, E. Kindombe-Luzolo, G. Ilombe, et al. A multidisciplinary investigation of the first chikungunya virus outbreak in matadi in the democratic republic of the Congo. *Viruses*, 13(10), 10 2021. ISSN 19994915. doi: 10.3390/v13101988.

M. Díaz-Menéndez, E. T. Esteban, M. Ujiie, G. Calleri, C. Rothe, D. Malvy, et al. Travel-associated chikungunya acquired in Myanmar in 2019. *Eurosurveillance*, 25(1), 1 2020. ISSN 15607917. doi: 10.2807/1560-7917.ES.2020.25.1.1900721.

G. B. Djeunang Dongho, G. Venturi, C. Fortuna, G. M. Paganotti, C. Severini, M. L'Episcopia, et al. Dengue and Chikungunya virus circulation in Cameroon and Gabon: molecular evidence among symptomatic individuals. *Access Microbiology*, 4(4), 4 2022. doi: 10.1099/acmi.0.000340.

P. Dudouet, P. Gautret, C. S. Larsen, M. Díaz-Menéndez, E. Trigo, F. von Sonnenburg, et al. Chikungunya resurgence in the Maldives and risk for importation via tourists to Europe in 2019–2020: A GeoSentinel case series. *Travel Medicine and Infectious Disease*, 36, 7 2020. ISSN 18730442. doi: 10.1016/j.tmaid.2020.101814.

F. Eyase, S. Langat, I. M. Berry, F. Mulwa, A. Nyunja, J. Mutisya, et al. Emergence of a novel chikungunya virus strain bearing the E1:V80A substitution, out of the Mombasa, Kenya 2017-2018 outbreak. *PLoS ONE*, 15(11 November), 11 2020. ISSN 19326203. doi: 10.1371/journal.pone.0241754.

F. Garwood. Fiducial Limits for the Poisson Distribution. *Biometrika*, 28(3/4):437, 12 1936. ISSN 00063444. doi: 10.2307/2333958.

D. Geleta, N. Tesfaye, and H. Ayigegn. Epidemiological Description of Chikungunya Virus Outbreak in Dire Dawa Administrative City, Western Ethiopia, 2019. *International Journal of Clinical and Experimental Medical Sciences*, 6(3):41, 2020. ISSN 2469-8024. doi: 10.11648/j.ijcems.20200603.13.

E. Javelle, S. A. Florescu, H. Asgeirsson, S. Jmor, G. Eperon, E. Leshem, et al. Increased risk of chikungunya infection in travellers to thailand during ongoing outbreak in tourist areas: Cases imported to europe and the middle east, early 2019. *Eurosurveillance*, 24(10), 3 2019. ISSN 15607917. doi: 10.2807/1560-7917.ES.2019.24.10.1900146.

A. B. Mayer, P. H. Consigny, M. P. Grobusch, D. Camprubí-Ferrer, R. Huits, and C. Rothe. Chikungunya in returning travellers from Bali - A GeoSentinel case series, 3 2023. ISSN 18730442.

C. Rizzo, S. T. Chin, E. van den Heuvel, and A. Di Bucchianico. Performance measures of discrete and continuous time-between-events control charts. *Quality and Reliability Engineering International*, 36(8):2754–2768, 12 2020. ISSN 10991638. doi: 10.1002/qre.2687.

V. Strassen. The Existence of Probability Measures with Given Marginals. *The Annals of Mathematical Statistics*, 36 (2):423–439, 4 1965. ISSN 0003-4851. doi: 10.1214/aoms/1177700153.

M. Torales, A. Beeson, L. Grau, M. Galeano, A. Ojeda, B. Martinez, et al. <i>Notes from the Field:</i> Chikungunya Outbreak — Paraguay, 2022–2023. *MMWR. Morbidity and Mortality Weekly Report*, 72(23):636–638, 6 2023. ISSN 0149-2195. doi: 10.15585/mmwr.mm7223a5.

F. Vairo, M. P. A. Coussoud-Mavoungou, F. Ntoumi, C. Castilletti, L. Kitembo, N. Haider, et al. Chikungunya Outbreak in the Republic of the Congo, 2019—Epidemiological, Virological and Entomological Findings of a South-North Multidisciplinary Taskforce Investigation. *Viruses*, 12(9), 9 2020. ISSN 19994915. doi: 10.3390/v12091020.

A. Wald. Sequential Tests of Statistical Hypotheses. *The Annals of Mathematical Statistics*, 16(2):117–186, 6 1945. ISSN 0003-4851. doi: 10.1214/aoms/1177731118.

© 2010 WILEY-VCH Verlag GmbH & Co. KGaA, Weinheim
